# Selective modulation of cell surface proteins during vaccinia infection: A resource for identifying viral immune evasion strategies

**DOI:** 10.1371/journal.ppat.1010612

**Published:** 2022-06-21

**Authors:** Delphine M. Depierreux, Arwen F. Altenburg, Lior Soday, Alice Fletcher-Etherington, Robin Antrobus, Brian J. Ferguson, Michael P. Weekes, Geoffrey L. Smith

**Affiliations:** 1 Department of Pathology, University of Cambridge, United Kingdom; 2 Cambridge Institute for Medical Research, University of Cambridge, United Kingdom; Ludwig-Maximilians-Universität München, GERMANY

## Abstract

The interaction between immune cells and virus-infected targets involves multiple plasma membrane (PM) proteins. A systematic study of PM protein modulation by vaccinia virus (VACV), the paradigm of host regulation, has the potential to reveal not only novel viral immune evasion mechanisms, but also novel factors critical in host immunity. Here, >1000 PM proteins were quantified throughout VACV infection, revealing selective downregulation of known T and NK cell ligands including HLA-C, downregulation of cytokine receptors including IFNAR2, IL-6ST and IL-10RB, and rapid inhibition of expression of certain protocadherins and ephrins, candidate activating immune ligands. Downregulation of most PM proteins occurred via a proteasome-independent mechanism. Upregulated proteins included a decoy receptor for TRAIL. Twenty VACV-encoded PM proteins were identified, of which five were not recognised previously as such. Collectively, this dataset constitutes a valuable resource for future studies on antiviral immunity, host-pathogen interaction, poxvirus biology, vector-based vaccine design and oncolytic therapy.

## Author summary

Vaccinia virus (VACV) is the vaccine used to eradicate smallpox and an excellent model for studying host-pathogen interactions. Many VACV-mediated immune evasion strategies are known, however how immune cells recognise VACV-infected cells is incompletely understood because of the complexity of surface proteins regulating such interactions. Here, a systematic study of proteins on the cell surface at different times during infection with VACV is presented. This shows not only the precise nature and kinetics of appearance of VACV proteins, but also the selective alteration of cellular surface proteins. The latter thereby identified potential novel immune evasion strategies and host proteins regulating immune activation. Comprehensive comparisons with published datasets provided further insight into mechanisms used to regulate surface protein expression. Such comparisons also identified proteins that are targeted by both VACV and human cytomegalovirus (HCMV), and which are therefore likely to represent host proteins regulating immune recognition and activation. Collectively, this work provides a valuable resource for studying viral immune evasion mechanisms and novel host proteins critical in host immunity.

## Introduction

Vaccinia virus (VACV) is a large, double-stranded (ds)DNA virus and is best known as the live vaccine used to eradicate smallpox [1]. Since smallpox eradication in 1980, research with VACV has continued because it is an excellent model to study host-pathogen interactions. Furthermore, VACV was developed as an expression vector [2,3] with utility as a vaccine against other infectious diseases [4–6] and as an oncolytic agent [7,8]. To optimise the design of VACV-based vaccines and oncolytic agents, it is important to develop a comprehensive understanding of the interactions between VACV-infected cells and the host immune system, and how VACV modulates these interactions. The study of virus-induced changes to cellular proteins has also led to several advances in understanding of host cell protein function. A recent example of this was our demonstration that histone deacetylase 4 (HDAC4) is degraded during VACV infection [9], is needed for the recruitment of STAT2 to the interferon (IFN)-stimulated response element during type I IFN-induced signalling and restricts the replication of VACV and herpes simplex virus type 1 [10].

VACV encodes many immunomodulatory proteins that function to evade or suppress the host immune response to infection [11]. Intracellular immunomodulators may inhibit innate immune signalling pathways, the activity of IFN-stimulated gene (ISG) products, or block apoptosis [12,13]. Secreted proteins can bind and inhibit cytokines, chemokines, IFNs or complement factors. Additional immunomodulators function on the cell surface to influence recognition of the infected cell by the immune system. In general, these have been studied less extensively than secreted or intracellular proteins. Nonetheless, it was reported that the viral haemagglutinin (HA, protein A56) modulates interactions with natural killer (NK) cells [14], and A40 is a cell surface protein with a type II membrane topology, with limited amino acid similarity to C-type lectins and NK cell receptors [15]. In addition to these integral membrane proteins, some VACV secreted proteins can also bind to the surface of infected or uninfected cells. Examples include the type I IFN-binding protein (B18 in VACV strain Western Reserve–WR) that binds to cell surface glycosaminoglycans [16,17] and the M2 protein that binds to B7.1 and B7.2 to prevent T cell activation [18,19]. The vaccinia complement control protein C3 (VCP) and the K2 serine protease inhibitor (serpin 3, SPI-3) each bind to A56 [20], and vaccinia epidermal growth factor (VGF) binds to the epidermal growth factor receptor and promotes cell division [21] and affects virus spread [22]. Other VACV proteins present on the infected cell surface are part of the outer envelope of the extracellular enveloped virus (EEV) [23].

In addition to VACV proteins expressed at the cell surface, there have been a few reports of changes to cellular plasma membrane (PM) proteins during infection. For instance, the abundance of different major histocompatibility complex class I (MHC-I) haplotypes, a major immune ligand, have been reported to change during infection and this might influence recognition of infected cells by both CD8^+^ cytotoxic T lymphocytes and NK cells [24–27]. However, changes in cell surface protein expression during VACV infection have not been addressed comprehensively or systematically.

This study used plasma membrane profiling (PMP) [28,29] to provide a comprehensive analysis of temporal and quantitative changes in host and viral proteins at the surface of immortalised human foetal foreskin fibroblasts (HFFF-TERTs) following VACV infection. Using tandem mass-tag (TMT)-based proteomics of PM-enriched fractions, >1000 PM proteins were quantified, and of these, 142 were downregulated and 113 were upregulated during infection. Twenty VACV proteins were detected at the cell surface including C8 and F5, which were not known to be present at the PM. Modulation of the expression levels of PM proteins indicated several possible novel immune evasion strategies, including selective downregulation of HLA-C and the IFN-α receptor 2 (IFNAR2) and upregulation of an apoptosis decoy receptor for TRAIL. The use of a proteasome inhibitor and comparison with previous studies by our group assessing whole cell protein expression during VACV infection [9] and protein stability [30] in HFFF-TERTs suggested that proteasomal degradation and host protein synthesis shut-off are not the major mechanisms by which PM proteins are downregulated during VACV infection, and that most PM proteins are likely upregulated through subcellular translocation and/or stabilisation at the PM. Finally, a comparative analysis with a dataset examining cell surface proteomic changes upon infection of HFFF-TERTs with human cytomegalovirus (HCMV) [31] identified possible common viral immune evasion strategies.

## Results

### Quantitative temporal analysis of the plasma membrane proteome during VACV infection

**Fig 1:**
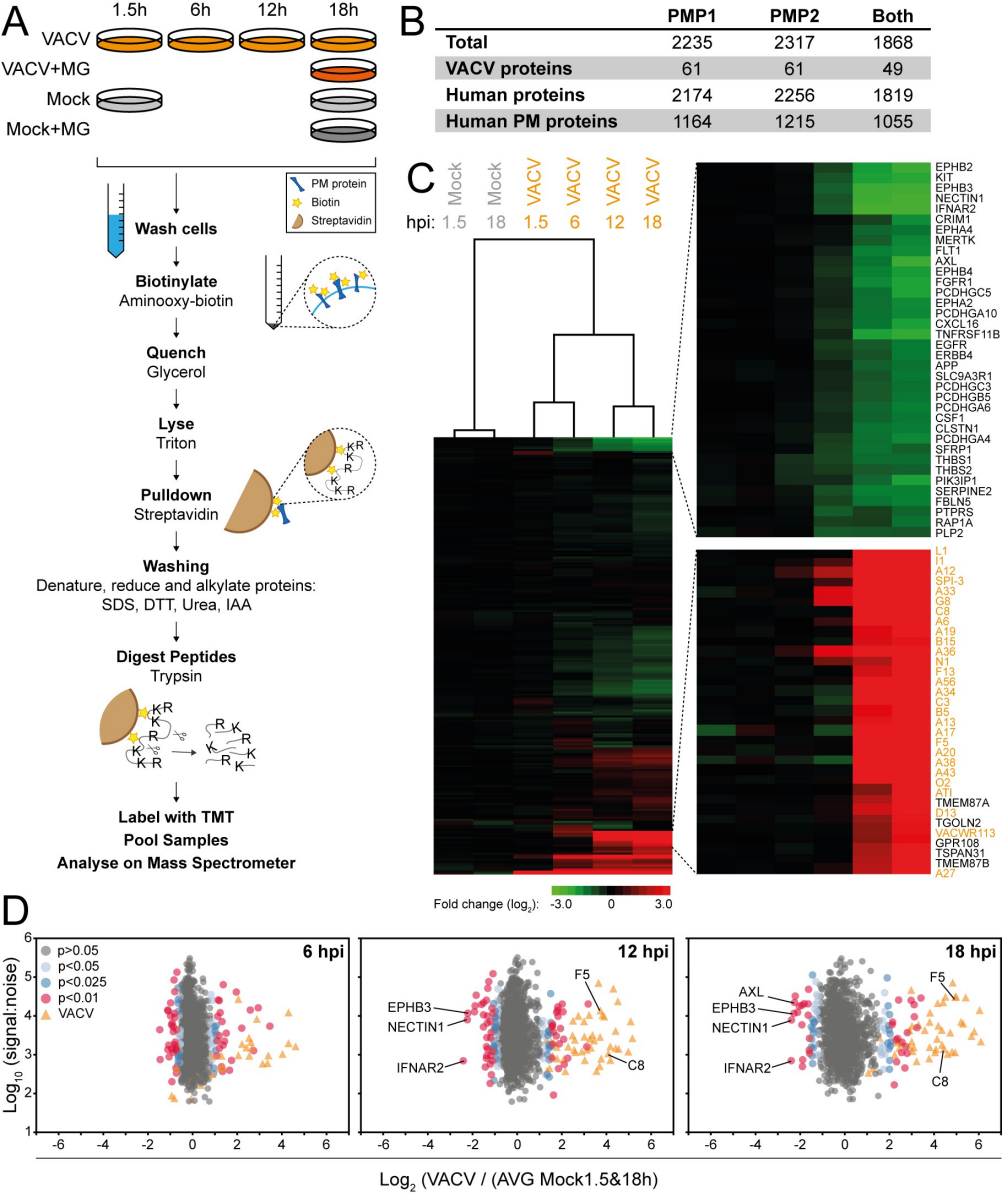
Quantitative temporal analysis of the plasma membrane proteome during VACV infection. (**A**) Schematic of the experimental workflow. HFFF-TERTs were mock-treated or infected with VACV at MOI 5 for the indicated time-points (S1A and S1B Fig). At 2 hpi MG132 was added to a mock and an infected sample (‘+MG’). Samples were generated in biological duplicate (PMP1, PMP2). (**B**) Number of proteins quantified in the PMP replicates. ‘Human PM proteins’ represents the number of proteins annotated with relevant GO terms (PM, ‘cell surface’ [CS], ‘extracellular’ [XC] and ‘short GO’ [ShG, 4-part term containing ‘integral to membrane’, ‘intrinsic to membrane’, ‘membrane part’, ‘cell part’ or a 5-part term additionally containing ‘membrane’]). (**C**) Hierarchical cluster analysis showing the fold change of all VACV and human proteins quantified in both replicates compared to mock (average 1.5 and 18 h, S1C Fig). Selected sections are shown enlarged and VACV proteins are indicated in orange. (**D**) Scatter plots of all VACV and human PM proteins quantified in both repeats at 6, 12 or 18 hpi. Selected human PM proteins were annotated. P-values were estimated using significance A with Benjamini-Hochberg correction for multiple hypothesis testing [117].

To measure how VACV infection changes the cell surface proteome, HFFF-TERTs were mock-treated or infected with VACV WR in biological duplicate. These cells were used previously in an investigation of the whole cell proteome during VACV infection by our group [9] and the whole cell lysate (WCL) and PM proteomes during HCMV infection [29], thereby enabling direct comparisons with these datasets. Additionally, a single mock and an infected sample were treated with the proteasome inhibitor MG132 at 2 hours post-infection (hpi). Flow cytometry confirmed that >95% of the cells were infected (S1A and S1B Fig). Multiplexed TMT and triple-stage mass spectrometry (MS3) were used to quantify the relative abundance of PM proteins at 1.5, 6, 12 and 18 hpi as described [28]. Briefly, infections were staggered so that the samples were washed simultaneously to remove excess and unattached virions. Surface sialic acids were oxidised and biotinylated with aminooxybioin, followed by quenching of the reaction and cell lysis. Biotinylated glycoproteins were then enriched, denatured and digested. The digested peptides from each sample were labelled with a different tandem mass tag (TMT), pooled and quantified by mass spectrometry (Fig 1A).

Human proteins were filtered for gene ontology (GO) annotations related to PM expression. Overall, 49 VACV proteins and 1055 human PM proteins were quantified in both experiments (Fig 1B). Mock-infected samples presented negligible variation in the abundance of any given protein over the course of the experiment (S1C Fig). VACV-infection induced selective changes in the expression of PM proteins, with the greatest fold-change (FC) occurring mostly late during infection (Fig 1C and 1D). This was reflected by separate clustering of mock samples, and samples harvested early (1.5 & 6 hpi) or late (12 & 18 hpi) after VACV infection (Fig 1C). All data are shown in S1 Table, in which the worksheet “Plotter” enables interactive generation of temporal graphs of the expression of each human or viral proteins quantified.

### Selective changes in human cell surface protein expression following VACV infection

**Fig 2:**
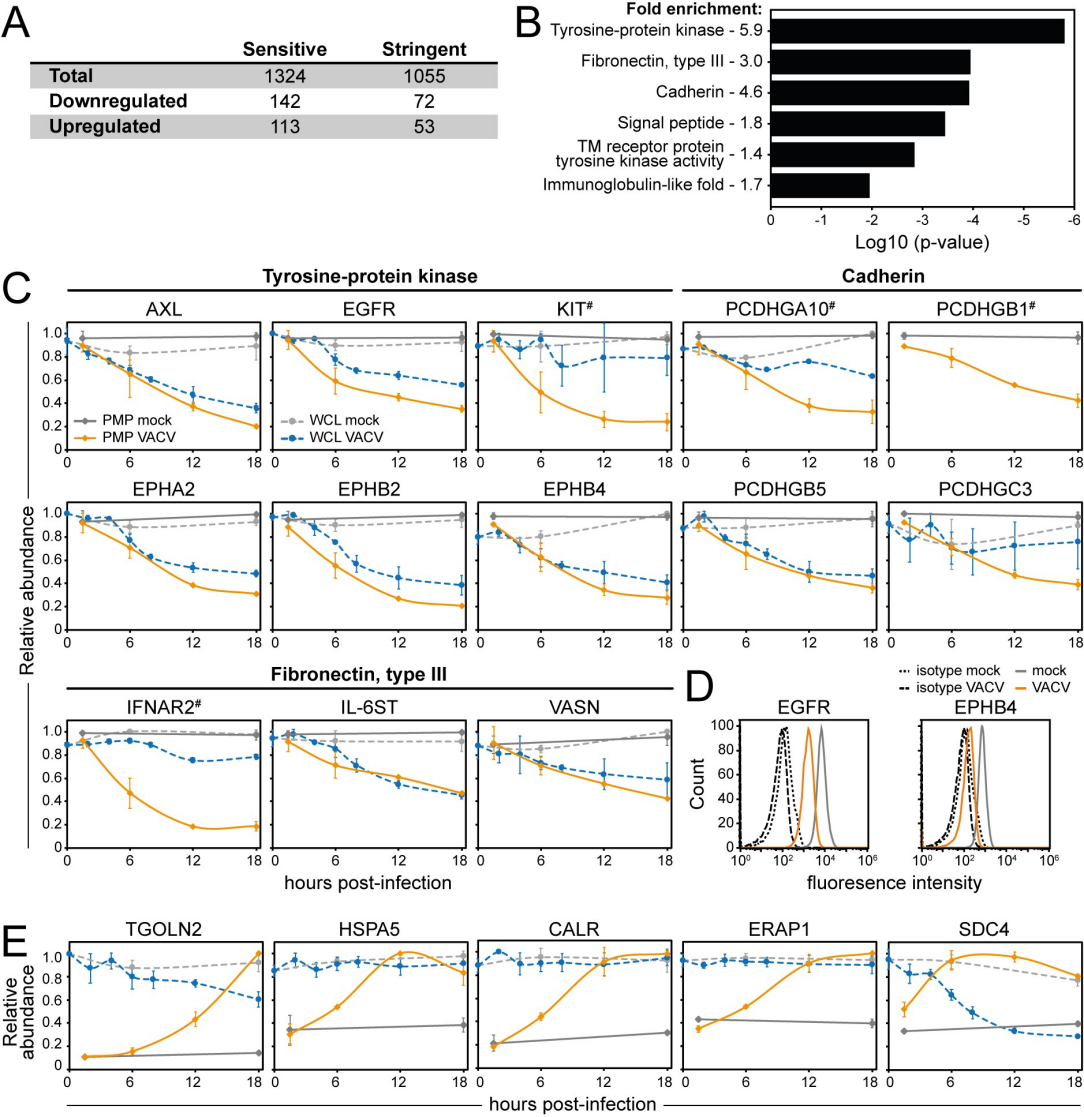
Selective modulation of host proteins at the cell surface during VACV infection. (**A**) Human host PM modulated according to the sensitive and stringent criteria (S2A–S2D Table). (**B**) DAVID functional enrichment of 142 proteins detected in either repeat and downregulated >2-fold. A background of all quantified human PM proteins was used. Representative terms are shown for each cluster with a Benjamini-Hochberg-corrected p-value <0.05 (S2E Table). (**C**) Temporal profiles of selected downregulated proteins, in which the fold-change for downregulation was in each case significant at p<0.05 (Benjamini-Hochberg-corrected one-way ANOVA, S2A and S2C Table). For each biological repeat, the sample with the highest quantitative value was set to 1.0, and the other samples were normalised accordingly. The plots show the mean+SD of the two repeats. (**D**) Downregulation of EGFR/EPHB4 during VACV infection was confirmed by flow cytometry at 15 hpi with VACV (MOI 5). Results are representative of 3 independent experiments. (**E**) Temporal profiles of selected upregulated proteins, all with p <0.05 (Benjamini-Hochberg-corrected one-way ANOVA, S2B and S2D Table). Data are represented as mean ± SD (PMP n = 2; WCL [9] n = 3, # WCL n<3).

Two sets of criteria were defined to determine which human PM proteins showed altered levels during VACV infection (Fig 2A and S2A–S2D Table). First, ‘sensitive’ criteria included proteins quantified in either or both PMP replicates showing >2-fold change (FC) at any time-point during infection. Second, ‘stringent’ criteria included only proteins detected in both PMP replicates showing >2 FC with a p-value <0.05 (Benjamini-Hochberg corrected one-way ANOVA). Both criteria indicated that VACV infection selectively alters the abundance of a small fraction (~1%) of human PM proteins detected in this study (Fig 2A and S2A–S2D Table). Sensitive criteria were used for subsequent analyses and proteins identified by stringent criteria are shown in S2 Table.

Of the 1055 human PM proteins quantified in both PMP replicates, 142 and 113 proteins were down- or upregulated, respectively (Figs 2A and S2A and S2B Table). The Database for Annotation, Visualization and Integrated Discovery (DAVID) [32,33] identified six functional clusters that were enriched within the group of downregulated human PM proteins (Fig 2B and S2E Table). This included protocadherins and several clusters associated with receptor tyrosine kinases (RTKs), which contain immunoglobulin domains (growth factor receptor families), fibronectin type III domains (ephrin family), or a combination of the two (TAM family) [34]. Temporal profiles provide insight into the kinetics of downregulation from the cell surface (this dataset) and as determined by WCL proteomics of VACV-infected cells [9] (Fig 2C). Downregulation of ephrin B4 (EPHB4) and epidermal growth factor receptor (EGFR) was confirmed by flow cytometry (Fig 2D).

DAVID functional enrichment analysis for the group of upregulated human PM proteins resulted in a single significantly enriched cluster: ‘Protein processing in the endoplasmic reticulum (ER)’ (S2F Table). The most highly upregulated proteins included many ER, Golgi and lysosomal proteins, such as trans-Golgi network integral membrane protein (TGOLN)2, heat shock protein (HSP)A5, calreticulin (CALR) and ER aminopeptidase (ERAP)1 (Fig 2E).

Interestingly, several host surface proteins involved with the cytokine response were modulated during VACV infection. For example, the interleukin-6 receptor subunit β (IL-6ST), interferon α/β receptor 2 (IFNAR2), mast/stem cell growth factor receptor Kit (KIT) (Fig 2C) and interleukin-10 receptor subunit β (IL-10RB, S1 Table) were substantially downregulated from the PM during VACV infection. Conversely, PM expression of several proteins involved in the suppression of the cytokine response, including CALR, ERAP1 and syndecan-4 (SDC4), were upregulated (Fig 2E). Overall, PM expression modulation of these proteins may indicate novel strategies by which VACV manipulates the cytokine environment to enhance immune evasion, replication or spread.

### Selective modulation of cell surface immune ligands during VACV infection

NK and T cells are essential components of the antiviral immune response. Their activation status is determined by the integration of inhibitory and activating signals emanating from receptors engaging with ligands expressed by (infected) target cells. Interestingly, several of these immune ligands showed altered PM expression during VACV infection.

**Fig 3:**
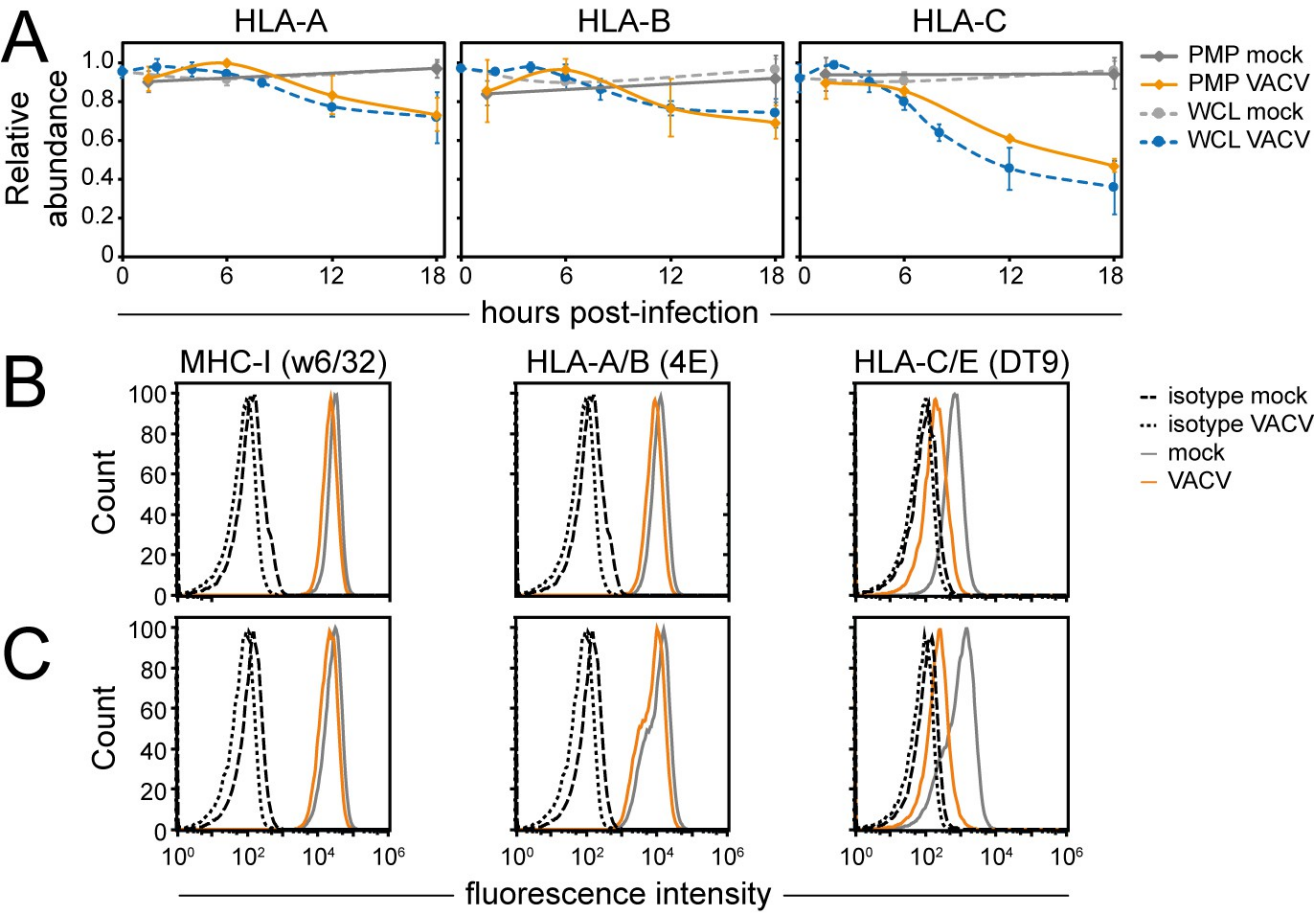
Selective downregulation of HLA-C from the PM during VACV infection. (**A**) Temporal profiles were generated only using peptides belonging uniquely to each of the indicated HLA-I heavy chains. Data are represented as mean ± SD (PMP n = 2; WCL [9] n = 3, # WCL n<3). (**B-C**) Cell surface downregulation of selected proteins during VACV infection (MOI 5) was confirmed by flow cytometry in HFFF-TERTs (**B**) or HeLa cells (**C**) at 15-18hpi. Results are representative of at least 2 independent experiments.

MHC-I (human leukocyte antigen class I (HLA-I) in humans) molecules are important regulators of NK and T cells. Due to the polymorphic nature of classical HLA-I (HLA-A, -B, -C), their peptides may easily be miss-assigned after detection by mass spectrometry. Therefore, only peptides corresponding uniquely to a single HLA-I type were included in this analysis. Interestingly, HLA-A and HLA-B were modestly downregulated whilst HLA-C was substantially downregulated (Fig 3A). This selective modulation was observed at both the cell surface and whole cell level and was further confirmed by flow cytometry in two different cell lines (Fig 3B and 3C). Given that all HLA-C subtypes are ligands for killer-cell immunoglobulin-like receptors (KIRs) expressed by NK cells, and less than 50% of the HLA-A/B subtypes can bind KIRs [35], these data suggest selective modulation of the NK cell response during VACV infection.

**Fig 4:**
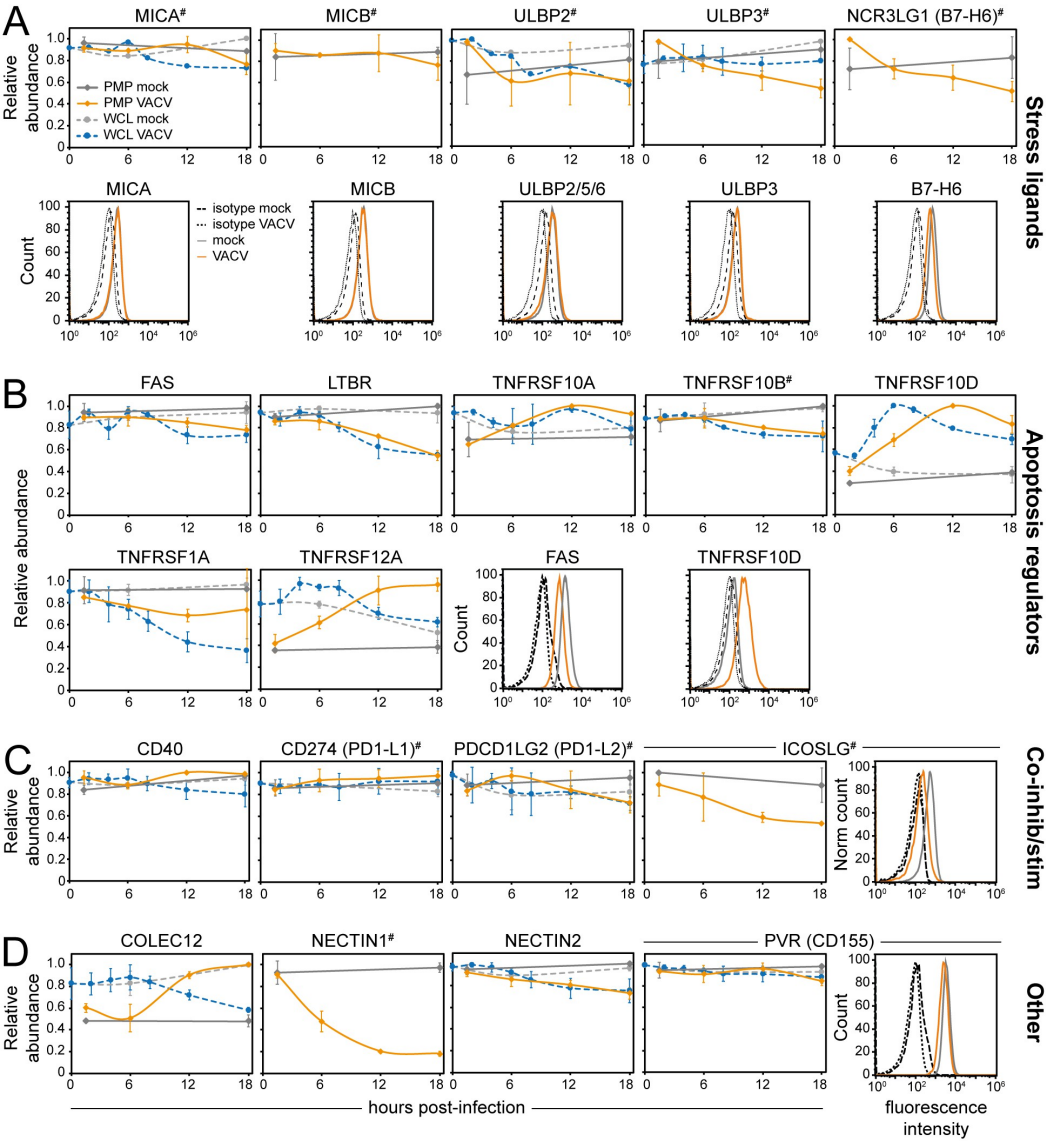
Cell surface expression of immune cell ligands is selectively modulated during VACV infection. Temporal profiles showing the cell surface and whole-cell expression levels of (**A**) stress ligands for NKG2D receptor, (**B**) apoptosis regulators, (**C**) co-inhibitory/stimulatory ligands and (**D**) other immune ligands. Data are represented as mean ± SD (PMP n = 2; WCL [9] n = 3, # WCL n<3). Cell surface downregulation of selected proteins during VACV infection was confirmed by flow cytometry in HeLa cells (stress ligands & TNFRSF10D), HFFF-TERTs (FAS & PVR) or DOHH2 cells (ICOSLG) at 15–18 hpi. Results are representative of at least two independent experiments.

Enhanced PM expression of stress molecules such as NK activating ligands HLA-I polypeptide related sequence (MIC)A/B, UL-16-binding proteins (ULBPS) and B7-H6 represents a conserved cellular response to stress, including viral infection [36–38]. However, the PM expression level of these proteins remained largely unchanged during VACV infection, which was confirmed by flow cytometry (Fig 4A). This may represent a new VACV strategy to evade immune recognition.

Selective modulation of regulators of lymphocyte-mediated apoptosis was observed during VACV infection. Downregulation of lymphotoxin-β receptor (LTBR) and tumour necrosis factor receptor superfamily member (TNFRSF) 1A (TNFR1), as well as upregulation of TNFRSF10D (TRAIL-R4), a decoy receptor for TNF-related apoptosis-inducing ligand (TRAIL), may lead to decreased sensitivity to apoptosis (Fig 4B). Upregulation of TNFRSF10D was confirmed by flow cytometry (Fig 4B). Conversely, upregulation of apoptosis inducer and NF-κB activator TNFRSF12A (Fn14) (Fig 4B) may sensitise the infected cell to lymphocyte-induced apoptosis. Expression levels of other surface proteins involved in apoptosis regulation, including FAS and TNFRSF10A/-B, remained largely unchanged (Figs 4B and S2).

Immune checkpoints are activating and inhibitory pathways that regulate the delicate balance between lymphocyte activation and maintenance of self-tolerance. During VACV infection, the levels of inhibitory checkpoint molecules programmed cell death ligand (PD-L)1, PD-L2 and B7-H3 were stable (Figs 4C and S2). The temporal profile of activating checkpoint molecule CD40 also remained mostly unchanged (Fig 4C). In contrast, repulsive guidance molecule B (RGMB), which has both stimulatory and inhibitory functions, was downregulated from the PM (S2E Fig). Additionally, inducible costimulatory ligand (ICOSLG) was downregulated from the cell surface, which was confirmed by flow cytometry (Fig 4C).

Other noteworthy changes in the surface proteome during VACV infection include the upregulation of collectin-1 (COLEC12), a ligand for the inhibitory NK receptor paired immunoglobulin-like type 2 receptor α (PILRα, S2F Fig). Furthermore, NECTIN-1, a ligand for the CD96 receptor with both inhibitory and stimulatory properties, was substantially downregulated. Other related proteins such as poliovirus receptor (PVR or CD155), NECTIN-2 and NECTIN-3 remained unchanged, suggesting that NECTIN-1 is targeted selectively by VACV. These changes may represent previously unrecognised NK cell immunomodulatory strategies employed by VACV. Conversely, modulation of surface expression levels of activating ligand vimentin (VIM, S2E Fig), may reflect the host antiviral response and enhance sensitivity to NK cell killing.

Lymphocytes rely on adhesion molecules to make contact with surrounding cells and determine whether they are targets to be eliminated. Six such molecules were quantified in the PMP replicates and showed only moderate downregulation for cadherin (CDH)2 and CDH4 (S2 Fig). Natural cytotoxicity receptor (NCR) ligands and CD47, a ligand for signal regulatory protein alpha (SIRP-α), remained largely unchanged during VACV infection (S2 Fig). Lastly, plexins, which are ligands for semaphorins, showed mild downregulation from the cell surface (S2 Fig).

**Fig 5:**
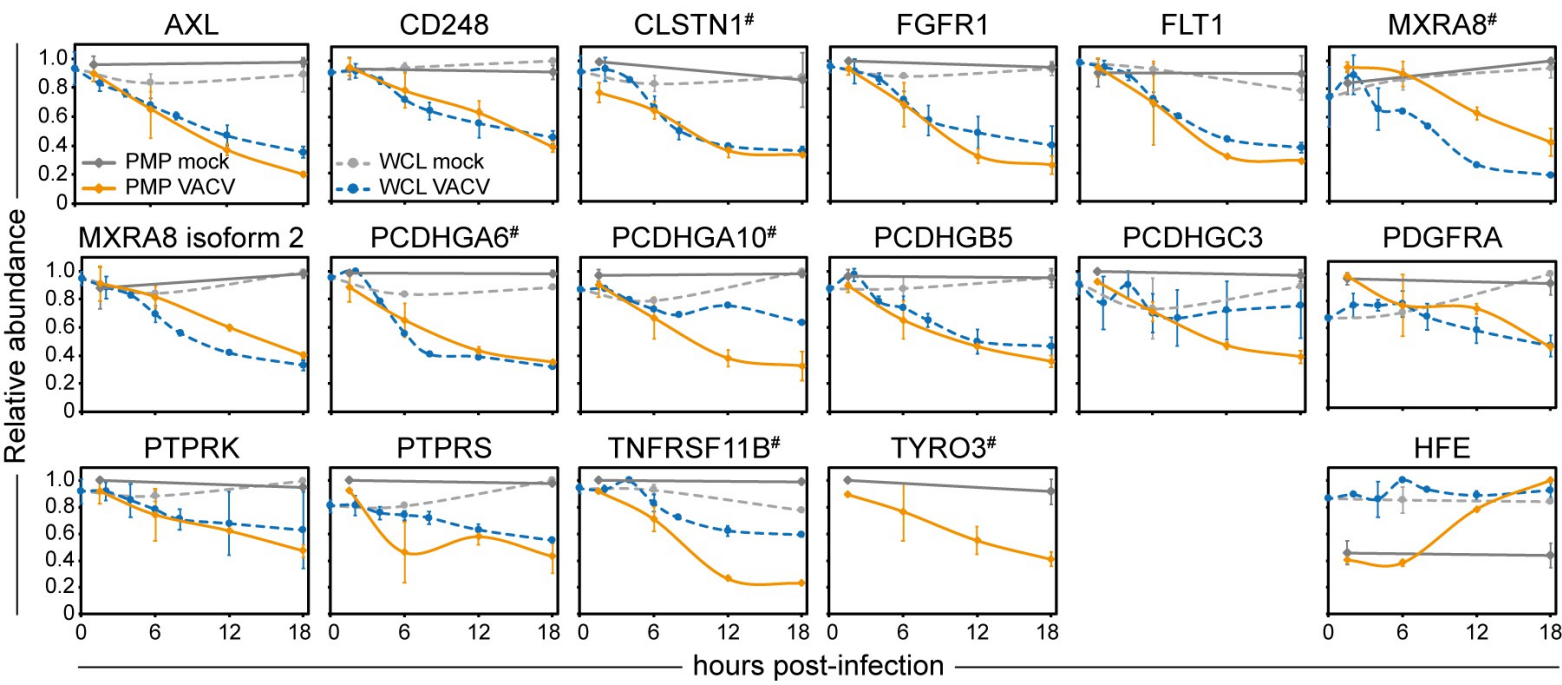
Modulation of surface expression of putative immune ligands during VACV infection. Temporal profiles of selected putative immune ligands modulated during VACV infection (S3 Table). Data are represented as mean ± SD (PMP n = 2; WCL n = 3 [9], # WCL < n = 3).

It is probable that not all receptor-ligand pairs involved in lymphocyte regulation have been identified. Most NK and T cell ligands display structural similarities and belong to a few protein families including cadherins, collagen, C-type lectin, TNF, HLA and immunoglobulin [39] and often these are modulated during viral infection. These characteristics were exploited to define putative candidate surface proteins with immunomodulatory functions. Host PM proteins substantially modulated during VACV infection were annotated with InterPro functional domains [40]. Six upregulated and 24 downregulated human PM proteins showed InterPro domain annotations associated with NK/T cell ligands, which may influence immune recognition (S3 Table). This included multiple protocadherins, endosialin (CD248), and several tyrosine-protein kinase receptors such as AXL, PTPRK, PTPRS and TYRO3 (Fig 5). Interestingly, protocadherins were also downregulated after infection with HCMV [30] and knockdown of protocadherin FAT1 in target cells led to decreased NK cell degranulation [29]. Additionally, FGFR1 was reported to co-stimulate T cells [41], and targeting of AXL sensitised lung cancer cells to lymphocyte-mediated cytotoxicity [42]. Taken together, these proteins modulated during VACV infection may represent putative immune ligands.

### VACV proteins detected at the plasma membrane

**Fig 6:**
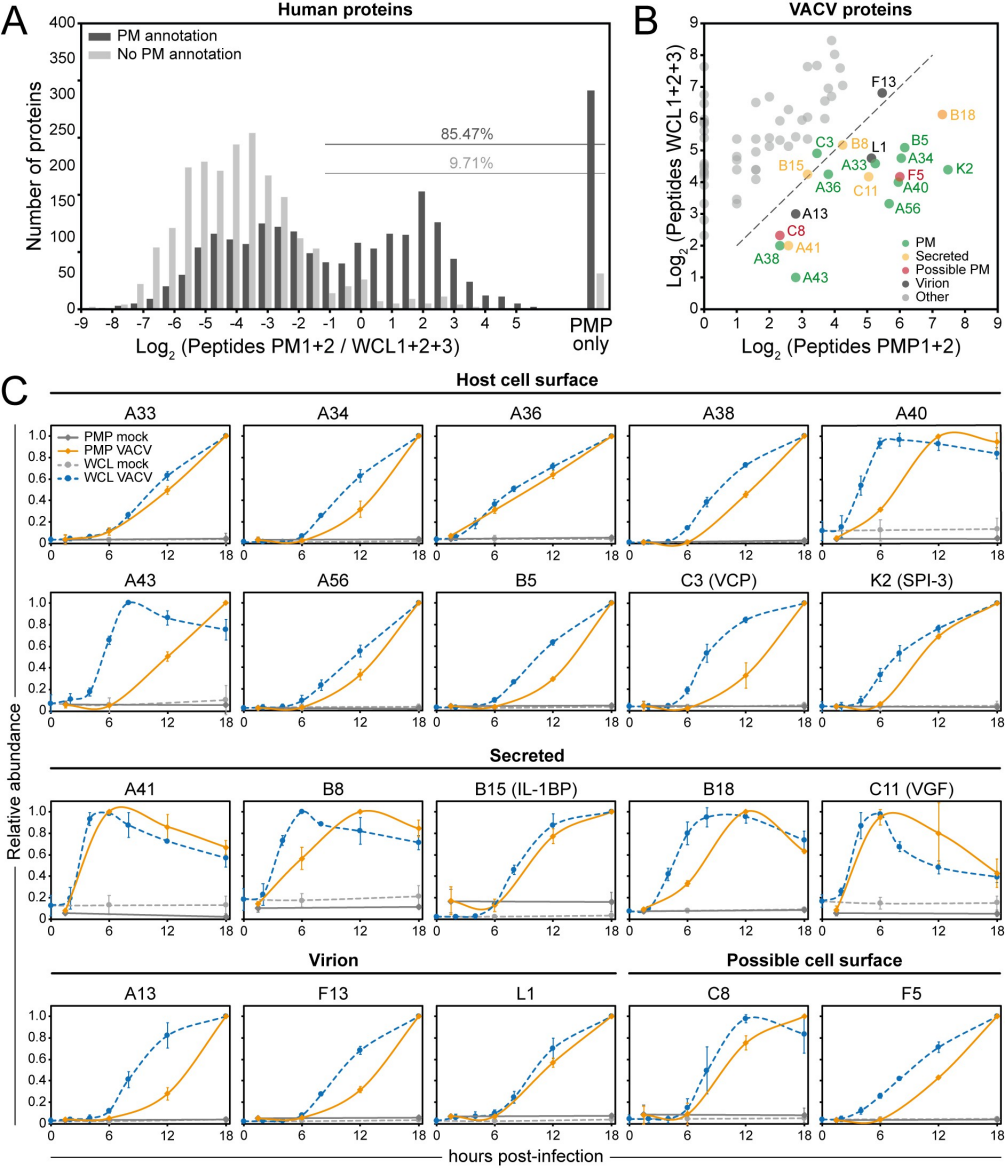
Identification of high-confidence VACV PM proteins. (**A**) Peptide ratios comparing the peptide count for a given protein in the PMP vs. WCL [9] for all human proteins quantified in both PMP replicates. ‘PMP only’ = not detected in any of the WCL replicates. ‘PM annotation’ includes GO terms PM, CS, XC and ShG. (**B**) Cut-off peptide ratio of 0.5 (dashed line), as determined in panel A, applied to 73 VACV proteins detected in either of the PMP replicates to identify high-confidence VACV PM proteins (Table 1). (**C**) Temporal profiles of all known and high-confidence VACV PM and secreted proteins. Data are represented as mean ± SD (PMP n = 2; WCL n = 3, A43: WCL n = 1).

The VACV proteins detected at the PM increased in number and abundance as infection progressed (Fig 1C and 1D). Given the incomplete annotation for the subcellular location of many VACV proteins, a filtering strategy was applied to discriminate between VACV proteins that are likely to be true PM proteins and non-PM contaminants that may have been detected due to, for example, their high intracellular abundance or disruption of EV virions. The number of peptides identified for a given protein was compared between PMP and WCL [9] proteomic datasets. For each human protein quantified in both PMP replicates, a peptide count ratio was calculated [29]: (peptide counts PMP 1+2) / (peptide counts WCL 1+2+3). More than 90% of the human proteins that were GO-annotated as non-PM showed a peptide ratio <0.5, whereas 85% of the proteins scoring above 0.5 were defined as human PM proteins (Fig 6A). This illustrates that the peptide ratio is a reliable metric to predict if a protein is likely to be expressed at the cell surface, or if it is a non-PM contaminant.

The peptide ratio of 0.5 was applied to the 73 viral proteins quantified in either PMP replicate, and this identified 20 VACV proteins as high-confidence PM proteins (Fig 6B and Table 1). Twelve of these are known to be present at the host cell surface (A33, A34, A36, A38, A40, A43, A56 [HA], B5, B18, C3 [VCP], C11 [VGF-1] and K2) and six are secreted proteins (A41, B8, B18, B15 [IL-1β-BP], C3 and C11) [43], illustrating the validity of the filtering strategy. Note that some of the secreted proteins are also retained at the cell surface. Five other VACV proteins were identified with high-confidence as PM proteins, including the structural proteins A13 and L1 that form part of intracellular mature virus (IMV) surface [44,45] and F13 that is present on the internal face of the outer membrane of the extracellular enveloped virus (EEV) [46, 47]. L1 and F13 had been identified on the cell surface following rupture of the EEV outer membrane [48]. The non-structural proteins C8 and F5 have not been described to interact with other VACV proteins and were identified as putative novel VACV PM proteins. F5 was reported to be located near the cell periphery, although has not been demonstrated previously to be exposed extracellularly [49].

**Table 1:** Details of high-confidence VACV PM proteins (related to Fig 6).

Uniprot	Gene name VACV-Cop	Gene name VACV-WR	Protein description / function**	Known location**	Temporal class *** [9]	Functional category [9,51]
**P01136**	C11L	VACWR009	C11, vaccinia growth factor, VGF	Secreted	1	Host interaction
**P24770**	B8R	VACWR190	B8, IFNγ-binding protein, gp	Secreted	1	Host interaction
**P24766**	A41L	VACWR166	A41, chemokine-binding protein, gp	Secreted	1	Host interaction
**P25213**	B19R	VACWR200 / B18R	B18, IFNα/β-binding protein, gp	Secreted & cell surface	2	Host interaction
**P24765**	A40R	VACWR165	A40, type II membrane, gp	PM	2	Host interaction
**P26671**	A43R	VACWR168	A43, gp	PM	2	Host interaction
**P18384**	K2L	VACWR033	K2, serine protease inhibitor 3 (SPI-3)	Secreted & PM	3	Host interaction
**P68619**	A36R	VACWR159	A36, protein egress and actin polymerisation	PM	3	Virion association (IEV only)
**P68638**	C3L	VACWR025	C3, vaccinia complement control protein (VCP)	Secreted & cell surface	3	Host interaction
**P17364**	C8L	VACWR020	C8	Unknown	3	Unknown
**P24761**	A34R	VACWR157	A34, EEV envelope, gp	PM	4	Virion association (EEV)
**Q01227**	B5R	VACWR187	B5, EEV envelope, gp	PM	4	Virion association (EEV)
**P24358**	F5L	VACWR044	F5, major membrane protein	Unknown	4	Unknown
**Q01218**	A56R	VACWR181	A56, haemagglutinin, EEV envelope, gp	PM	4	Virion association (EEV)
**P68617**	A33R	VACWR156	A33, EEV envelope, gp	PM	4	Virion association (EEV)
**Q76ZQ4**	A13L	VACWR132	A13, IMV membrane protein	IMV	4	Virion association (IMV)
**P24763**	A38L	VACWR162	A38, gp	PM	4	Host interaction
**P25212**	B16R *	VACWR197 / B15R	B15, IL-1β-binding protein	Secreted	4	Host interaction
**P07612**	L1R	VACWR088	L1, IMV membrane protein	IMV	4	Virion association (IMV)
**P04021**	F13L	VACWR052	F13, EEV membrane-associated protein	EEV	4	Virion association (EEV)

Gene names from VACV strain Copenhagen (Cop) have L or R to indicate direction of transcription. IEV = intracellular enveloped virus. IMV = intracellular mature virus. EEV = extracellular enveloped virus. gp = glycoprotein.

*Gene non-functional in VACV strain Copenhagen.

**For a review of VACV protein function and location see [43].

***Temporal classes 1 and 2 occur before viral DNA replication.

VACV protein expression has been categorised into four temporal classes [9,50–54]. The high-confidence VACV PM proteins cover the four temporal classes, although the majority are expressed late during infection (temporal profile (Tp) 4, Table 1). Overall, the expression kinetics of the VACV proteins showed a slight delay in PM expression compared to the whole cell, which may reflect the time required for protein transport to the cell surface (Fig 6C). Interestingly, the temporal profiles of some secreted VACV proteins showed a reduction in abundance at late time-points, consistent with protein secretion (Fig 6C). Notably, the appearance of proteins C3 (VCP), and K2 (SPI-3) at the PM closely matched the kinetics of cell surface A56 (HA) to which C3 and K2 bind [20].

### Mechanisms underlying changes in human PM protein expression during VACV infection

**Fig 7:**
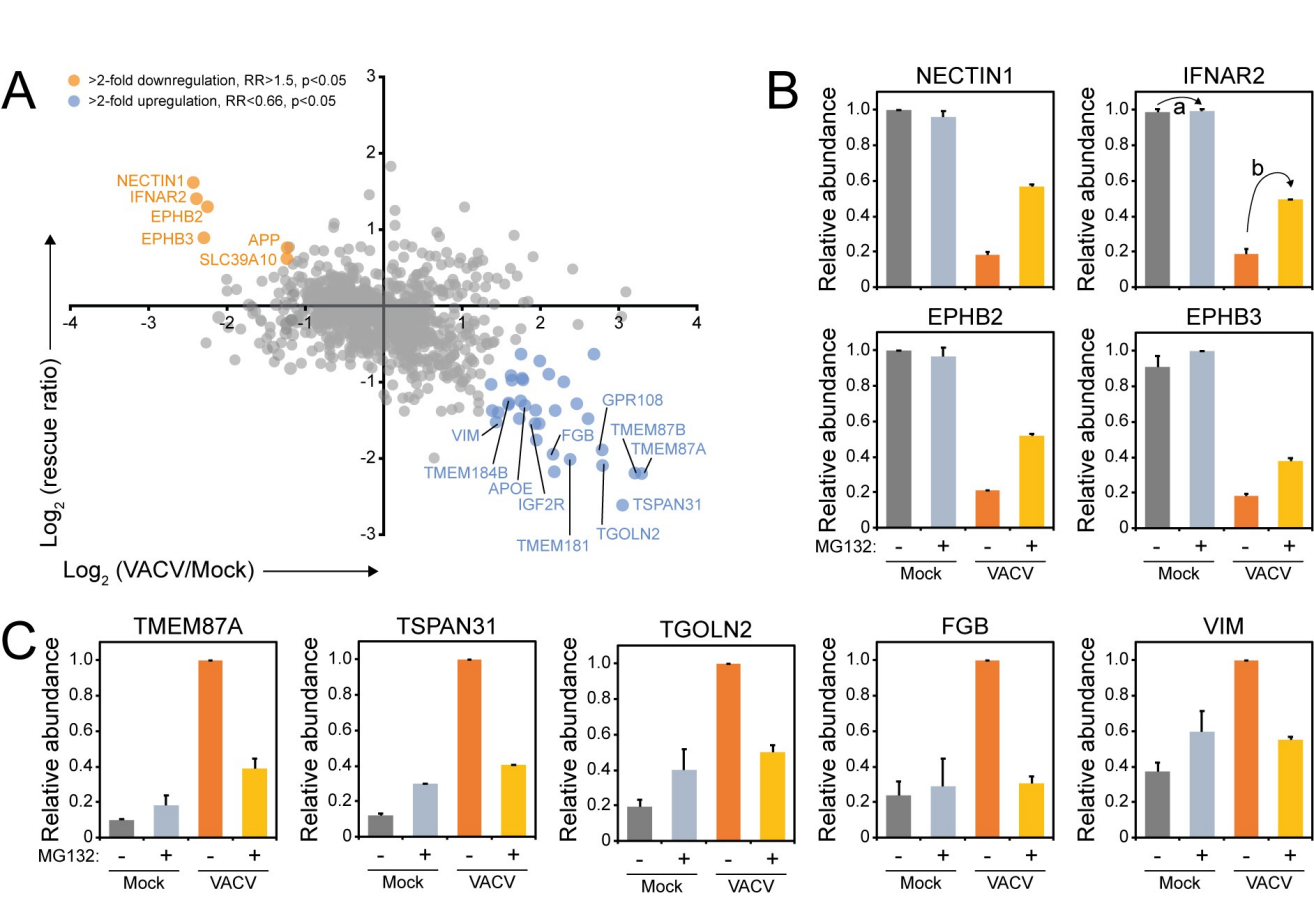
Systematic analysis of proteasome-dependent changes in surface protein expression during VACV infection. (**A**) Identification of human PM proteins downregulated from the cell surface at 18 hpi (compared to 18 h mock) in both replicates and rescued by addition of MG132 (>2 FC, rescue ratio (RR) >1.5, p<0.05), or upregulated at the cell surface at 18 hpi (compared to 18 h mock) and diminished by the addition of MG132 (>2-FC, RR<0.66, p<0.05) (S4 Table). Here, we define the RR = b / a, where a = protein abundance during VACV infection +MG132 / abundance during infection -MG132. This value was limited to 1 to avoid artificial ratio inflation. b = protein abundance during mock-treatment +MG132 / abundance during mock -MG132 (see panel B, IFNAR2). P-values were estimated using significance A with Benjamini-Hochberg correction for multiple hypothesis testing [117]. (**B**) Relative abundance of selected human proteins downregulated at the PM at 18 hpi and rescued by addition of MG132. (**C**) Relative abundance of selected human PM proteins for which upregulation was prevented by the addition of MG132 at 18hpi. Data are represented as mean ± SD (n = 2) S1 Table.

The expression of PM proteins during viral infection can be modulated by several mechanisms such as proteasomal/lysosomal degradation, arrest of synthesis or protein translocation. MG132 inhibits proteasomal degradation but also affects lysosomal cathepsins [55]. To identify which PM proteins are modulated by active degradation, a VACV-infected and a mock sample were treated with MG132. MG132 was added at 2 hpi to allow the uncoating of VACV that relies on the proteasome [56–58]. An MG132 rescue ratio (RR) was calculated by comparing the abundance of a given protein during VACV infection ±MG132 with the abundance of the same protein during mock-treatment ±MG132. Of the 73 proteins downregulated >2-fold in both replicates at 18 hpi, six (8.2%) showed a RR >1.5 (Fig 7A and 7B and S4A Table). EPHB3 and APP also showed a RR >1.5 in the WCL MG132 analysis [9] (S1 Table). NECTIN1, INFAR2 and EPHB2 were not detected in the WCL MG132 dataset. Taken together, unlike the WCL proteins, cell surface protein downregulation is likely regulated predominantly through mechanisms other than proteasomal degradation.

Interestingly, addition of MG132 modulated cell surface expression of approximately a third of the human PM proteins upregulated during VACV infection (Fig 7A and 7C and S4B Table). In this and previous studies, it was observed that addition of MG132 inhibits expression of late VACV genes, but not early genes [9,57,58]. Therefore, proteasome-dependent upregulation of proteins at the cell surface may indicate that a late VACV protein is responsible for the observed increase in expression. Alternatively, these proteins may normally be retained inside the cell by a second host protein which is degraded by the proteasome during VACV infection, resulting in upregulation at the cell surface.

VACV is known to shut-off host protein synthesis [59–63]. Consequently, proteins with a short half-life may be downregulated from the PM during VACV infection due to natural turnover. In a previous study, protein turnover in HFFF-TERTs was quantified over 18 h by pulse (p)SILAC and 730 human PM proteins were identified [30]. The abundance of 12 host proteins downregulated >2-fold from the cell surface during VACV infection was also shown to be reduced >2 fold in the pSILAC study (S3 Fig and S4C Table). Taken together, these data suggest that proteasomal degradation and host protein synthesis shut-off are not the major mechanisms by which PM proteins are downregulated during VACV infection.

Host PM expression can be modulated during viral infection by degradation or enhanced production, but also by a translocation mechanism such as secretion, shedding, enhanced intracellular recycling or intracellular trapping. To identify human PM proteins that are up/downregulated during VACV infection via a translocation mechanism, protein expression levels in the PMP and WCL datasets were compared. The WCL dataset [9] was filtered for human proteins quantified in any of the replicates and showing on average >2 FC at any time-point (2, 4, 6, 8, 12, 18 hpi) compared to 18 h mock sample to determine which proteins are up-/downregulated during VACV infection. Proteins that showed altered surface expression in this PMP study but were not detected in the WCL study include the upregulated proteins CLN3, CD68 and TMEM219, and downregulated proteins EPHA8, NECTIN1, PIK3IP1, CXCL16 and protocadherins PCDHB11 and PCDHGB1.

**Fig 8:**
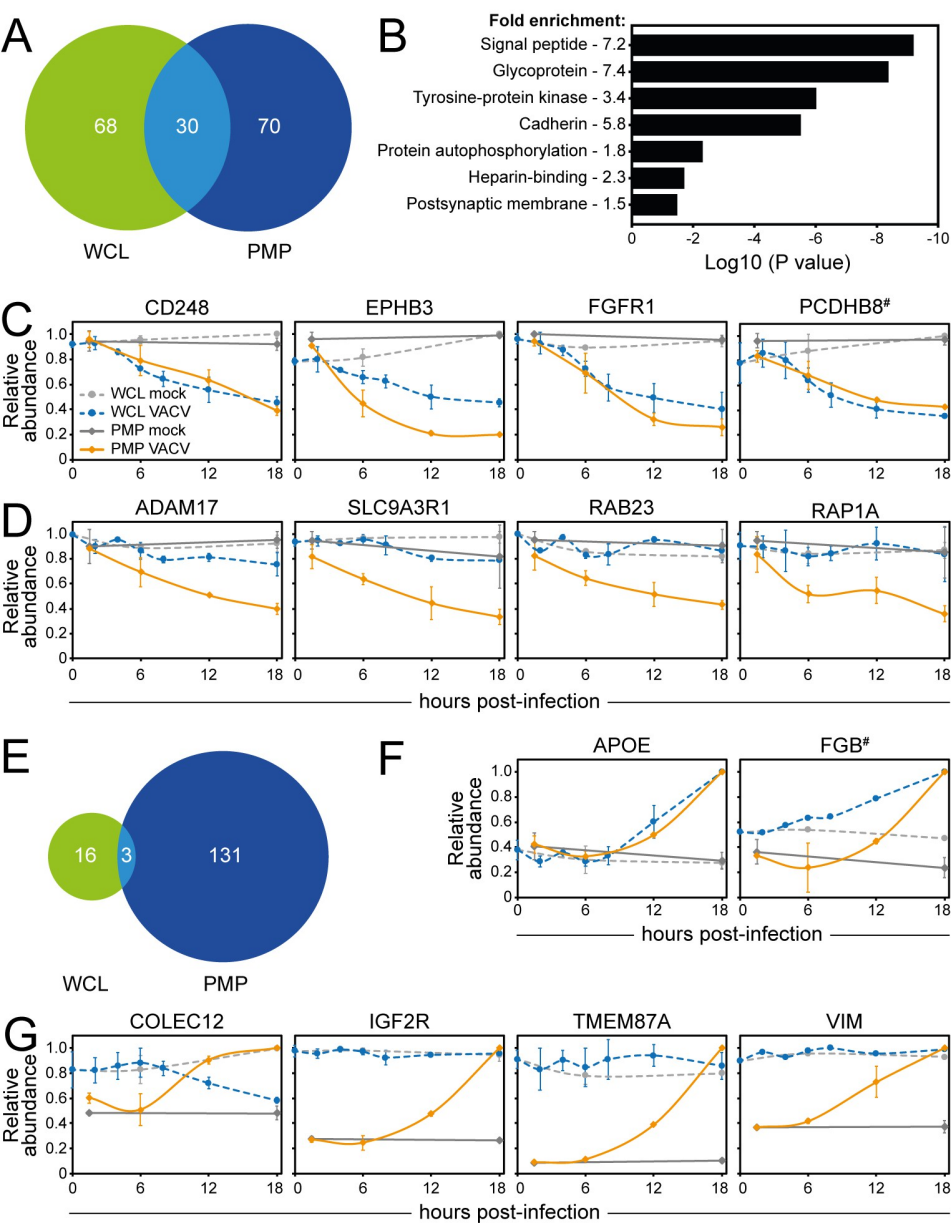
Cell surface-specific regulation of protein expression during VACV infection. (**A**) Overlap of downregulated proteins between PMP and WCL according to ‘sensitive’ criteria (S5A and S5B Table). ‘Sensitive’ criteria WCL (n = 3) [9]: human proteins quantified in any of the replicates and showing on average >2-fold-change at any time-point (2, 4, 6, 8, 12, 18 hpi) compared to 18 h mock sample. (**B**) DAVID functional enrichment of the 30 proteins commonly downregulated from the cell surface and at whole-cell levels. A background of all proteins detected in PMP and WCL proteomics was used. Representative terms from each cluster with a Benjamini-Hochberg-corrected p-value of <0.05 are shown (S5C Table). (**C**) Temporal profiles of selected host proteins that were commonly downregulated from the cell surface and at whole-cell level. (**D**) Temporal profiles of selected human PM proteins that were only downregulated at the cell surface, but not at whole-cell level (**E**) Same as panel A using proteins upregulated according to ‘sensitive’ criteria (S5D Table). (**F**) Temporal profiles of selected human PM proteins that were commonly upregulated from the cell surface and at whole-cell level. (**G**) Temporal profiles of selected human PM proteins that are only upregulated at the cell surface, but not at whole-cell level. Data are represented as mean ± SD (PMP n = 2; WCL n = 3 [9], # WCL n<3).

Eighty-eight point five percent of the proteins downregulated >2-fold from the cell surface during VACV infection were also quantified in our prior WCL proteomic analysis, representing a total of 100 proteins. Within this group, about a third were downregulated in both studies (Fig 8A and S5A and S5B Table). DAVID functional enrichment analysis of these proteins showed significant enrichment of functional clusters, including ‘Tyrosine-protein kinase’, ‘Protein autophosphorylation’, ‘Cadherin’, ‘Heparin-binding’ and ‘Postsynaptic membrane’ (Fig 8B and S5C Table). This includes the ephrin protein family, IL6-ST and several, but not all, protocadherins (Figs 2C and 8C). An additional ~20 human PM proteins downregulated in the PMP dataset showed a downward trend in the WCL but did not meet the cut-off criteria (e.g. EGFR or protocadherin gamma (PCDHG) A10, Fig 2C). The remainder of the proteins were downregulated solely at the PM, indicating internalisation without active degradation (e.g. IFNAR2 and KIT, Fig 2C).

Ninety-four point four percent of the human proteins >2-fold upregulated at the cell surface were also detected in the WCL proteomics, representing 134 proteins. Only three of these proteins were upregulated at both the cell surface and whole cell level during VACV infection: apolipoprotein E (APOE), TNFRSF10D and fibrinogen β-chain (FGB, Figs 4B and 8E–8G and S5D Table). This is suggestive of enhanced protein synthesis, despite host shutoff. However, most upregulated proteins, including TGOLN2, HSPA5, CALR, ERAP1, SDC4, TNFRSF12A, COLEC12 and VIM, were only upregulated at the cell surface, indicating translocation to and/or stabilisation at the surface (Figs 2E,4B and 8F and S5E Table).

### Identification of human PM proteins commonly targeted during virus infections

**Fig 9:**
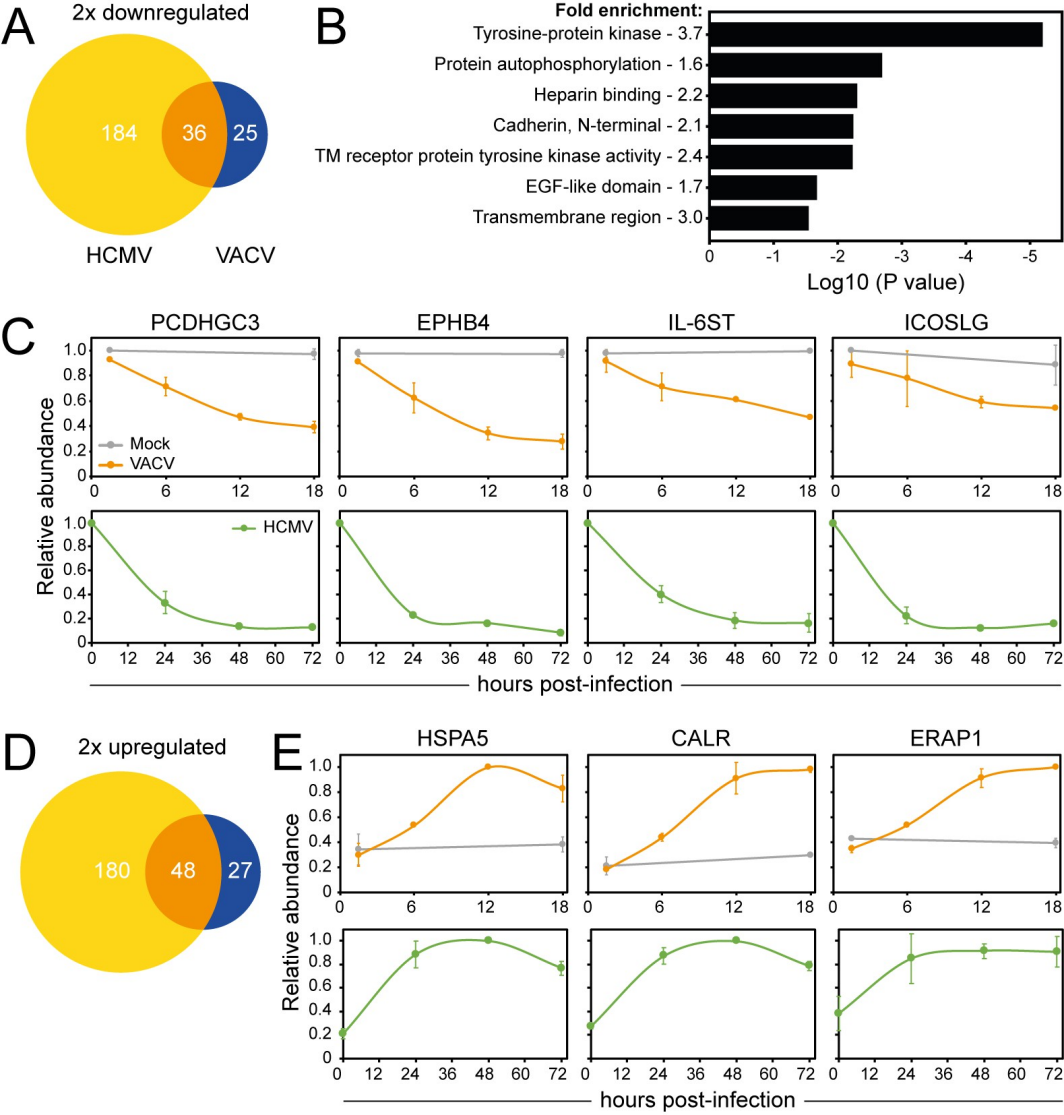
HCMV and VACV commonly target a subset of PM proteins. (**A**) Overlap of proteins downregulated according to ‘sensitive’ criteria after infection with VACV or HCMV (S6A Table). ‘Sensitive’ criteria HCMV PMP (n = 2) [29]: human PM- (GO terms PM/CS/XC/ShG) proteins quantified in a single or both replicates and showing on average >2 FC at any time-point (24, 48, 72 hpi) compared to (average of) mock sample(s). (**B**) Functional enrichment within proteins commonly downregulated from cell surface during VACV or HCMV infection (‘sensitive’ criteria). A background of all proteins detected in at least one replicate of both PMP VACV and PMP HCMV was used. Shown are representative terms from each cluster with a Benjamini-Hochberg-corrected p-value of <0.05 (S6B Table). (**C**) Temporal profiles of selected proteins commonly downregulated from the cell surface after VACV or HCMV infection. (**D-E**) As panel A/C, respectively, using proteins upregulated by ‘sensitive’ criteria (S6C Table).

Host proteins that play an important role in antiviral immunity are often targeted by multiple viruses. To identify these proteins, the PMP dataset was compared to a published dataset analysing the cell surface proteome during infection with another dsDNA virus, HCMV [29,64]. Given the different replicative niche of VACV and HCMV, common targets of these two viruses are of particular interest. Human PM proteins quantified in either or both replicates and showing on average >2 FC at any time-point (24, 48, 72 hpi compared to the average of mock samples) were considered as up-/downregulated during HCMV infection (S6 Table). VACV infection led to a more selective modulation of protein expression compared to HCMV, but a considerable overlap between PM proteins up- or downregulated during VACV or HCMV infection was observed (Fig 9A and 9D and S6A and S6C Table).

Fifty-four percent of the proteins downregulated at the cell surface during VACV infection were quantified in the HCMV PMP dataset. DAVID analysis of the commonly downregulated proteins revealed that both viruses target RTKs—EPHB3, EPHA4, EPHA2, EPHB4, ERBB2, PDGFRA, FLT1, ERBB4, EPHB2 -, molecules with EGF-like domains—THBS2, NID2, FBLN5, CD248, FBLN1, THBS1, VASN -, and cadherins—PCDHGB5, PCDHGA10, PCDHGC5, PCDHGC3 (Fig 9B and S6B Table). Other noteworthy proteins downregulated by both viruses include IL-6ST and ICOSLG, indicating that these proteins may be important in the antiviral response (Fig 9C).

Fifty-two point eight percent of the proteins upregulated at the PM during VACV infection were quantified in the HCMV PMP dataset. DAVID functional enrichment of proteins upregulated at the cell surface by both VACV and HCMV infection revealed enrichment of functional clusters for ‘Protein processing ER’ and ‘Prevents secretion ER’. These clusters likely relate to ER stress, which is commonly triggered during viral infections due to a substantial increase in protein production, or present novel evasion strategies [65–67]. Several of the PM proteins that were strongly upregulated during VACV infection were also upregulated at the cell surface during HCMV infection, including HSPA5, CALR and ERAP1 (Fig 9E and S6C Table).

## Discussion

In this study, quantitative temporal plasma membrane proteomics was used to assess systematically the impact of VACV infection on host and viral PM protein expression. Altered PM protein expression may represent a normal response to viral infection, VACV-mediated suppression of immune responses and/or modulation of the environment to support virus production and spread.

VACV infection resulted in selective modulation of host PM protein expression. Notably, substantial downregulation of several members of the RTK and protocadherin families was observed and these protein families were also downregulated from the PM during HCMV infection [29]. Several members of the RTK (FGFR1, PDGFRA, KIT, FLT1, AXL, TYRO3) and protocadherin (PCDHGA10, PCDHGA6, PCDHGB5, PCDHGB7, PCDHGC3) families contain InterPro functional domains often found in immune ligands [39,68], suggesting that they may act as immune regulators. This hypothesis is supported by previous reports that PM expression of the protocadherin FAT1 leads to decreased degranulation of NK cells [29] and that the RTK ephrin B2 leads to T cell co-stimulation [69]. Particularly notable was the downregulation of the RTK EGFR, which was also observed after HSV-1 and HCMV infection [29,70,71]. EGFR downregulation from VACV-infected cells is of particular interest because VACV also expresses a viral epidermal growth factor (called vaccinia growth factor, VGF, protein C11) that contributes to virulence [72]. VGF stimulates cells surrounding the infected cell to proliferate, causing hyperplasia and mitotic bodies characteristic of orthopoxvirus pathology [21]. More recently, VGF was also reported to enhance motility of infected cells to promote viral spread [22]. The removal of EGFR from the surface of VACV-infected cells may represent a strategy to promote binding of VGF to EGFR on surrounding uninfected cells thereby stimulating the metabolic activity of these cells to enhance virus replication. Alternatively, the removal of EGFR from the infected cell surface may reflect internalisation of activated EGFR upon engagement with VGF, followed by degradation rather than recycling to the PM [73].

PM proteins upregulated during VACV infection included chaperone proteins whose translocation to the PM is associated with ER stress and activation of the immune system [74]. Some of these proteins are manipulated by viruses to suppress immune responses. For example, CALR suppresses IFN-α production and antiviral activity in the context of hepatitis B virus infection [67], ERAP1 induces cleavage of cytokine receptors [65,66], and syndecan-4 (SDC4) negatively regulates retinoic acid-inducible gene I (RIG-I)-mediated signalling during virus infection [75]. Overall, the increased PM expression of these proteins during VACV infection may indicate previously unknown strategies by which VACV manipulates the host response to infection.

VACV is well-known to interfere with cytokine signalling by expressing soluble binding proteins, or decoy receptors, for IL-1β, TNFα, IL-18, IFN-ɣ and IFN-α/β [11]. In addition, IFN signalling is targeted at several levels in the pathway downstream of the receptor [76,77]. The observed downregulation of IFNAR2 (type I IFN receptor) and IL10-RB (a component of multiple cytokine receptors, including type III IFNs) from the PM, may represent novel VACV strategies for evasion of the IFN response.

NK and T cells are activated by changes on the surface of the infected cell. There are contested reports that VACV infection leads to the mild downregulation of total HLA-I surface levels [14, 24–27]. However, the selective downregulation of HLA-C from the cell surface observed here is consistent with a previous study relying on HLA transfection [27]. HLA-A and -B represent the majority of cell surface HLA-I [78], which explains why reduction of HLA-C did not substantially affect the total HLA-I levels detected by flow cytometry. HLA subtypes have a differential impact on the immune response and whilst most HLA-C molecules are KIR ligands, HLA-A and -B mostly interact with T cell receptors. Importantly, KIRs and HLA polymorphisms are linked to infectious disease outcome [79] and if the selective modulation of HLA-C is conserved in other orthopoxviruses, such as variola virus, this might have contributed to pathogenesis of smallpox.

The activating NKG2D ligands and B7-H6 (NKp30 ligand), which are typically upregulated in response to viral infection or in cancer cells [26,80], were not upregulated during VACV infection and this may represent a novel strategy by which VACV evades the NK cell response. In contrast, VIM, a ligand for the activating receptor NKp46, was upregulated at the PM during VACV infection, whilst the total cell level remained unchanged. VIM upregulation sensitises mycobacterium tuberculosis-infected cells to NKp46-mediated lysis [81], facilitates adenovirus type 2 transport [82] and interacts with VACV virions and facilitates their assembly [83]. Taken together, this suggests that translocation of VIM to the cell surface during VACV infection may be caused by virion transport. The immune system may have evolved a strategy to detect this through NKp46-mediated recognition of VIM.

Further, several immune checkpoints were selectively modulated during VACV infection. PD-L1 was not affected during VACV infection, which is in contrast with HCMV-induced up-regulation of PD-L1 [29]. Downregulation of the costimulatory molecule ICOSLG potentially represents a novel mechanism by which VACV modulates NK and T cells [84–87]. Additionally, RGMB downregulation may interfere with T cell costimulatory function [88] or affect co-inhibitory pathways [89]. The impact of modulation of these proteins on the immune response remains to be determined.

More VACV proteins were detected at the PM than had been described hitherto, and a filtering strategy was used to distinguish likely true viral PM proteins from those that might represent ‘overspill’ from an abundant intracellular pool. This process identified 5 possible new PM proteins: IMV envelope proteins A13 and L1 [44,45], the EEV outer membrane protein F13 [46] and non-structural proteins C8 and F5. L1, A13 and F13 might either be expressed at the PM or alternatively, their detection there might result from interactions with other proteins. For example, the presence of F13 might reflect its interaction with B5 and A56 [90,91]. C8 and F5 are not present in IMV particles [92–94] and are not known to interact with other VACV proteins [95]. F5 has a transmembrane domain and is expressed at the periphery of the infected cell, in regions in contact with neighbouring cells [49]. The subcellular localisation of C8 is unknown, but it also contains a hydrophobic domain that might function as either a signal peptide or transmembrane domain. Overall, these findings justify further study of the roles of C8 and F5 during VACV infection, particularly since non-structural PM proteins may have additional roles in immune regulation.

Systematic comparison of the PMP dataset with various other datasets gave insight into mechanisms used to regulate cell surface protein expression during VACV infection. Only six downregulated PM proteins were rescued by addition of MG132 (8.2%), which contrasts with the WCL proteomics where 69% of the proteins were rescued by MG132 [9]. Nevertheless, most of the proteins downregulated from the PM were also downregulated, or showed a downward trend, at the whole-cell level. A combination of VACV-induced host shut-off and high protein turnover may explain the downregulation of a few proteins. However, most downregulated human PM proteins are likely degraded through non-proteasomal mechanisms, for example, lysosomal degradation. The remainder of the proteins were downregulated specifically from the cell surface, and not at the whole-cell level, likely indicating internalisation and/or retention in intracellular compartments without active protein degradation.

Proteins from the same family were not always regulated by the same mechanism. For example, the downregulation of PM ephrin B2 and B3, but not other ephrins, was prevented by addition of MG132. Additionally, most protocadherins were downregulated in both PMP and WCL experiments, however, protocadherin-γ A10 and C3 did not show a clear downregulation at whole-cell level. Furthermore, protocadherin-γ B4 downregulation may be the result of a high protein turnover in combination with host shut-off by VACV. This may indicate that VACV has developed several mechanisms to modulate specific members of a protein family, which may have particularly important functions as novel immune ligands.

Strikingly, even though HCMV and VACV encode a similar number of genes, VACV modulates the abundance of fewer proteins compared to HCMV. Similar observations were made after comparing WCL datasets of VACV or HCMV infection [9]. The greater alteration of host protein abundance by HCMV, may reflect the longer and more complex HCMV infectious cycle including the ability to enter and exit the nucleus and establish latency. Nonetheless, there is considerable overlap between proteins targeted by both viruses. Targeting of the whole protocadherin family by multiple viruses may indicate that these proteins represent previously unknown immune ligands.

Fifty-eight percent of proteins quantified in both replicates PMP1 and PMP2 were determined to be of PM origin based on Gene Ontology annotation. The remainder of the proteins are likely to be a mixture of: (a) non-PM contaminants. We find that when fractionating peptide samples in order to perform in-depth analysis of PM proteins, the percentage of proteins annotated as having a PM origin is somewhat reduced in comparison to unfractionated samples, which typically exhibit 70–90% purity [28,96]. The increased identification of non-PM contaminants seen following high resolution fractionation suggests that contaminants form a minor part of the total protein content and highlights the efficiency of PM protein enrichment. Some of these contaminants might include proteins endogenously biotinylated at low level such as histones [97]. (b) Genuine PM proteins that have not yet been annotated as such. (c) Proteins that interact with high affinity with PM proteins yet do not actually have extracellular domains. Previously we found that the fraction of peptides that have been directly biotinylated (glycopeptides eluted from streptavidin beads using PNGaseF) is generally of very high purity [96]. This, in addition to data demonstrating the membrane impermeability of both aminooxy-biotin and the oxidation reagent sodium periodate [98] suggests that non-PM proteins are nevertheless infrequently labelled or enriched.

It is difficult to know how many PM proteins are potentially ‘missing’ from the dataset, since the complete proteome of human fibroblasts is unknown. Possibly the best ways to estimate this number are (a) by comparison to previous PM datasets from human fibroblasts. In our prior analysis of the PM proteome of HFFFs during infection with HCMV, up to 1184 PM proteins were quantified [29], similar to the 1164–1215 proteins quantified in the present study. Of note, given the considerable technological improvements in the present study, both in terms of the method used for peptide fractionation and in terms of the sensitivity of the mass spectrometer (Orbitrap Fusion Lumos versus Orbitrap Elite), the relatively modest increase in the number of quantified PM proteins may suggest that ~1,200 PM proteins is indeed the limit of detection for the HFFF PM proteome. (b) By comparison to our data from whole cell lysate proteomic analysis of HFFF-TERTs infected with VACV [9]. By adding Gene Ontology annotations to this whole cell data, an estimate of the maximum possible number of PM proteins can be obtained, which is 1692. However, it is unclear how many of these proteins are actually expressed at the PM. Furthermore, some may be expressed at an insufficient level to enable quantification, even by the sensitive mass spectrometry methods employed. Additionally, some may have small ectodomains rendering detection by our strategy unfeasible.

The PMP technique detects changes in expression levels, rather than modifications to proteins. Therefore, immune evasion strategies involving masking, interference with receptor-ligand binding and/or molecular mimicry may not be identified using this approach. Furthermore, the use of HFFF-TERTs–allowing for direct comparison with previously published data with the same cell type [9, 29, 30, 64]–limited the detection of immune ligands that are more commonly found on professional antigen presenting cells. Nonetheless, a number of potentially novel immunomodulatory strategies by VACV were identified. Overall, the PMP dataset represents a valuable resource providing new research avenues informing on antiviral immune responses and viral immune evasion strategies, vaccine vector design and oncolytic virus therapy.

## Materials & methods

### Cell lines

Primary HFFF immortalised with human telomerase (HFFF-TERTs) [99] were grown in Dulbecco’s modified Eagle’s medium (DMEM; Gibco, Thermo Fisher Scientific, Lutterworth, UK) supplemented with 10% foetal bovine serum (v/v; Seralab, London, UK) and 1% penicillin/streptomycin (p/s; Gibco, Thermo Fischer, UK). BSC-1 (African green monkey cell line, ATCC CCL-26) were grown in DMEM supplemented with 10% filtrated bovine serum (FBS; Pan Biotech UK Limited, Dorset, UK) and 1% P/S. HeLa cells (human cervical ATCC CCL-2) and RK13 (Rabbit kidney cells, ATCC CCL37) were grown in Minimum Essential Medium (MEM; Gibco, Thermo Fischer, Lutterworth, UK) supplemented as described above for BSC-1 cells. Non-Hodgkin’s B cell line DOHH2 (kind gift from Dr Daniel Hodson) was grown in Roswell Park Memorial Institute (RPMI)-1640 medium (Gibco, Thermo Fischer, Lutterworth, UK) with 10% FBS and 1% p/s. All cell lines were maintained at 37°C in 5% CO_2_. Cells were routinely checked as mycoplasma negative (MycoAlert, Lonza, UK).

### Vaccinia virus

VACV strain Western Reserve (WR) stocks were produced by infection of RK13 cells. Virus particles were released from cells by three freeze-thaw cycles and two rounds of 20 strokes of a Dounce homogenizer. Cell-free viruses were resuspended in 10 mM Tris HCl pH 9.0 and purified by sedimentation through a 36% (w/v) sucrose cushion twice (Fisher Chemical, Thermo Fisher Scientific). Purified viruses were resuspended in PBS, titrated in BSC-1 cells by plaque assay and stored at -80°C.

### Virus infections and MG132 treatment

Immediately before infection, the culture medium on 15 cm^2^ dishes with 4–6.5 × 10^6^ HFFF-TERTs was replaced with infection medium (DMEM supplemented with 2% FBS and 1% p/s). Cells were mock-treated or infected at MOI 5 with VACV in 6 ml infection medium. After 90 min adsorption at 37°C, 15 ml of infection medium was added, and samples were incubated at 37°C until harvesting. For samples treated with the proteasome inhibitor MG132, 0.5 μl/ml medium (v/v; Merck, Kenilworth, U.S.A.) of 20 mM MG132 was added per flask at 2 hpi. Infections were staggered to allow for simultaneous harvesting of the indicated time-points.

### Plasma membrane profiling and TMT labelling

Plasma membrane protein labelling was performed as described [29]. Briefly, cell surface sialic acid residues were oxidised and biotinylated using 1 mM sodium periodate (Thermo Scientific), 100 mM aminooxy-biotin (Biotium Inc., Fremont, U.S.A.) in dry DMSO and 10 mM aniline (Sigma-Aldrich, Merck, Dorset, U.K.) in PBS pH 6.7 (Sigma-Aldrich). After 30 min at 4°C, the reaction was stopped by addition of glycerol (Sigma-Aldrich) at a final concentration of 1 mM. Cells were harvested into 1.6% Triton X-100 (Fisher Scientific), 150 mM NaCl (Sigma-Aldrich), 5 mM iodoacetamide (Sigma-Aldrich) in 10 mM Tris-HCl pH 7.6 (Sigma-Aldrich) supplemented with protease inhibitor tablets (Roche, Merck). Biotinylated glycoproteins were enriched with high-affinity streptavidin agarose beads (Pierce) and washed extensively. Captured protein were denatured with SDS and urea, reduced with DTT, alkylated with iodoacetamide (IAA, Sigma) and digested on-bead with trypsin (Promega) in 200 mM HEPES (4-(2-hydroxyethyl)-1piperazineethanesulfonic acid) pH 8.5 for 3 h. The digested peptides were eluted, and each sample labelled with 56 μg of a unique TMT reagent (Thermo Fisher Scientific) in a final acetonitrile concentration of 30% (v/v) for 1 h at room temperature. Samples were labelled as follows. For replicates PMP1 and PMP2: TMT 126 (WT VACV 90 min), TMT 127N (WT VACV 6 h), TMT 127C (WT VACV 12 h), TMT 128N (WT VACV 18 h), TMT 128C (Mock 18 h), TMT 130N (WT VACV + MG132 18 h), TMT 130C (Mock + MG132 18 h), TMT11-131C (Mock 90 min). For replicate PMP3: TMT 127C (WT VACV 18h), TMT11-131C (Mock). The reaction was quenched with hydroxylamine to a final concentration of 0.5% (v/v). TMT labelled samples were combined at equal ratio, vacuum-centrifuged and subjected to C18 solid-phase extraction (Sep-Pak, Waters).

### HpRP Fractionation and LC-MS3

An unfractionated single-shot sample was analysed to ensure similar peptide loading across each TMT channel. The remaining TMT-labelled tryptic peptide samples were subjected to HpRP fractionation, as described [30] except that the samples were prepared as a single-set of 6 fractions. The fractions were dried and resuspended in 10 μl MS solvent (4% MeCN/5% formic acid) prior to LC-MS3. Data from the single-shot experiment was analysed with data from the corresponding fractions to increase the overall number of peptides quantified. Mass spectrometry data was acquired using an Orbitrap Lumos (Thermo Fisher Scientific, San Jose, CA). An Ultimate 3000 RSLC nano UHPLC equipped with a 300 μm ID x 5 mm Acclaim PepMap μ-Precolumn (Thermo Fisher Scientific) and a 75 μm ID x 50 cm 2.1 μm particle Acclaim PepMap RSLC analytical column was used. Loading solvent was 0.1% FA, analytical solvent A: 0.1% FA and B: 80% MeCN + 0.1% FA. All separations were carried out at 40°C. Samples were loaded at 5 μL/min for 5 min in loading solvent before beginning the analytical gradient. The following gradient was used: 3–7% B over 3 min, 7–37% B over 173 min, followed by a 4-min wash at 95% B and equilibration at 3% B for 15 min. Each analysis used a MultiNotch MS3-based TMT method [100,101]. The following settings were used: MS1: 380–1500 Th, 120,000 Resolution, 2 × 10^5^ automatic gain control (AGC) target, 50 ms maximum injection time. MS2: Quadrupole isolation at an isolation width of m/z 0.7, CID fragmentation (normalised collision energy (NCE) 35) with ion trap scanning in turbo mode from m/z 120, 1.5 x 10^4^ AGC target, 120 ms maximum injection time. MS3: In Synchronous Precursor Selection mode the top 10 MS2 ions were selected for HCD fragmentation (NCE 65) and scanned in the Orbitrap at 60,000 resolution with an AGC target of 1 × 10^5^ and a maximum accumulation time of 150 ms. Ions were not accumulated for all parallelisable time. The entire MS/MS/MS cycle had a target time of 3 s. Dynamic exclusion was set to +/- 10 ppm for 70 s. MS2 fragmentation was trigged on precursors 5 × 10^3^ counts and above.

### Protein quantification and data processing

Mass spectra were processed as described in [9] using MassPike, a sequest-based software pipeline, through a collaborative arrangement with Professor Steven Gygi’s laboratory (Harvard Medical School). A combined database was constructed from (i) the human UniProt database (26^th^ January 2017), (ii) the VACV strain WR UniProt database (23^rd^ February 2017), (iii) common contaminants such as porcine trypsin. The combined database was concatenated with a reverse database composed of all protein sequences in reversed order. Searches were performed using a 20 ppm precursor ion tolerance, product ion tolerance was set to 0.03 Th. TMT tags on lysine residues and peptide N termini (229.162932 Da) and carbamidomethylation of cysteine residues (57.02146 Da) were set as static modifications, while oxidation of methionine residues (15.99492 Da) was set as a variable modification. To control the fraction of erroneous protein identifications, a target-decoy strategy was employed [102,103]. Peptide spectral matches (PSMs) were filtered to an initial peptide-level false discovery rate (FDR) of 1% with subsequent filtering to attain a final protein-level FDR of 1% [104,105]. PSM filtering was performed using a linear discriminant analysis, as described [106]. This distinguishes correct from incorrect peptide IDs in a manner analogous to the widely used Percolator algorithm [107], though employing a distinct machine learning algorithm. The following parameters were considered: XCorr, DCn, missed cleavages, peptide length, charge state, and precursor mass accuracy. Protein assembly was guided by principles of parsimony to produce the smallest set of proteins necessary to account for all observed peptides [106]. Proteins were quantified by summing TMT reporter ion counts across all matching peptide-spectral matches using “MassPike,” as described [100, 101]. A minimum one unique or shared peptide per protein was used for quantitation. Briefly, a 0.003 Th window around the theoretical m/z of each reporter ion (126, 127n, 127c, 128n, 128c, 129n, 129c, 130n, 130c, 131n, 131c) was scanned for ions, and the maximum intensity nearest to the theoretical m/z was used. The primary determinant of quantitation quality is the number of TMT reporter ions detected in each MS3 spectrum, which is directly proportional to the signal-to-noise (S:N) ratio observed for each ion [108]. Conservatively, every individual peptide used for quantitation was required to contribute sufficient TMT reporter ions (minimum of 1375 per spectrum) so that each on its own could be expected to provide a representative picture of relative protein abundance [100]. Additionally, an isolation specificity filter was employed to minimize peptide co-isolation [109]. Peptide-spectral matches with poor quality MS3 spectra (more than 9 TMT channels missing and/or a combined S:N ratio of less than 275 across all TMT reporter ions) or no MS3 spectra at all were excluded from quantitation. Peptides meeting the stated criteria for reliable quantitation were then summed by parent protein, in effect weighting the contributions of individual peptides to the total protein signal based on their individual TMT reporter ion yields.

Protein quantitation values were exported for further analysis in MS Excel. For protein quantitation, reverse and contaminant proteins were removed, then each reporter ion channel was summed across all quantified proteins and normalised assuming equal protein loading across all channels. Fractional TMT signals (i.e., reporting the fraction of maximal signal observed for each protein in each TMT channel, rather than the absolute normalised signal intensity) was used for further analysis and to display in figures. This effectively corrected for differences in the numbers of peptides detected per protein. For all proteins quantified in the PMP screens, normalized S:N ratio values are presented in S1 Table (‘Data original’ (PMP1 & 2) & ‘repeat 3 18h’ (PMP3) worksheets). For PMP1 & 2, peptide sequences initially assigned to HLA-A, -B or -C were manually compared to reference sequences of classical HLA-I expressed by HFFF-TERTs (HLA-A11:01, -A24:02, -B35:02, -B40:02, -C02:02, and -C04:01) [110]. Only the peptides matching uniquely to the reference sequence of a single subtype (-A, -B, -C) were included. The summed S:N values of these peptides was used for the relative abundance of HLA-A, HLA-B or HLA-C and are available in S1 Table (‘Data’ worksheet).

### Validation of infection and modulation of host protein expression levels by flow cytometry

In parallel with infections for PMP, 10 cm^2^ dishes with ~1x10^6^ cells were mock-treated or infected with VACV at MOI 5. At 15.5 hpi, cells were harvested using trypsin-EDTA (Gibco) and fixed and permeabilised using Fixation/Permeabilization Solution Kit according to the manufacturer’s instructions (BD Biosciences, San Jose, U.S.A.). Cells were stained with a mouse anti-D8 monoclonal antibody AB1.1 [111] followed by PE-conjugated goat anti-mouse IgG (BioLegend, San Diego, U.S.A.). To assess human PM protein expression levels, HFFF-TERTs or HeLa cells were mock-treated or infected with VACV at MOI 5 in DMEM 2% FCS, 1% p/s. Cells were harvested at 14–16 hpi cells using accutase solution as per the manufacturer’s instructions (Sigma-Aldrich). ICOSLG surface expression could not be detected by flow cytometry on HFFF-TERTs, therefore, DOHH2 cells were infected at MOI 50 with VACV in RPMI-1640 with p/s. At 1 hpi, RPMI-1640 supplemented with 2% FBS (v/v) and 1% p/s was added to resuspend cells at 500,000 cells / 100 μl. All validation of PM protein expression was performed with 2–2.5x10^5^ cells / well, stained with Zombie NIR/Violet Fixable Viability Kit (Biolegend) in combination with an antibody against HLA-I (W6/32, kindly provided by Dr L.H. Boyle), MICA (2C10, Santa Cruz Biotechnology, Santa Cruz, CA, USA), EPHB4 (rea923, Miltenyi Biotech, Bergisch Gladbach, Germany), CD95 (DX2, Miltenyi Biotech), EGFR (528, Santa Cruz), HLA-B/C (4E, kindly provided by Dr L.H. Boyle), HLA-C/E (DT9, kindly provided by Dr L.H. Boyle), ULBP3 (166510, R&D systems), B7-H6 (875001, R&D systems, Minneapolis, U.S.A), ULBP-2/5/6 (65903, R&D systems), Plexin-B1 (rea728, Miltenyi Biotech), MICB (236511, R&D systems), trail-R4 (104918, R&D systems), APC-conjugated ICOSLG Monoclonal Antibody (MIH12), isotype mouse Igg2b (Santa Cruz), isotype mouse IgG2a (Santa Cruz) isotype mouse IgG1 (Santa Cruz), Mouse IgG1 kappa Isotype Control (P3.6.2.8.1) conjugated with APC (eBioscience–part of Thermo Fisher Scientific, San Diego, CA, USA). Samples stained with unconjugated primary antibodies were subsequently probed with PE-conjugated Goat anti-mouse IgG (BioLegend). Cells were fixed using Fixation/Permeabilization Solution Kit according to the manufacturer’s instructions (BD Biosciences). All samples were analysed by flow cytometry using the Invitrogen Attune NxT and FlowJo software.

### Data analysis and bioinformatics

Analyses were performed using original python code using NumPy [112], pandas [113], Matplotlib [114], SciPy.stats [115] and sklearn.metrics [116] packages. To describe human PM proteins in this study, host proteins were filtered for relevant gene ontology (GO) annotations indicative of PM location; PM, ‘cell surface’ [CS], ‘extracellular’ [XC] and ‘short GO’ [ShG, 4-part term containing ‘integral to membrane’, ‘intrinsic to membrane’, ‘membrane part’, ‘cell part’ or a 5-part term additionally containing ‘membrane’]. Two sets of criteria were defined to determine which human PM proteins showed modulated expression levels during VACV infection. First, ‘sensitive’ criteria included proteins quantified in either or both PMP replicates showing >2x FC at any time-point during infection. Second, ‘stringent’ criteria limited the false discovery rate further and included proteins detected in both PMP replicates showing >2x FC with a p-value <0.05 (Benjamini-Hochberg corrected one-way ANOVA). To include all biologically relevant proteins, ‘sensitive’ criteria were used for subsequent analyses. A hierarchical clustering analysis based on uncentered Pearson correlation was performed on the FC with Cluster 3.0 (Stanford University). To avoid skewing the data with outliers, the FC was limited to 50 in either direction. A heatmap was visualised using Java TreeView (Fig 1C). Volcano plots in Fig 1D do not show VACV protein H7 because there was no quantitative value available in the mock sample. Scale of the x-axis of these plots was limited from -7 to 7, which excluded VACV proteins C6 and RPO7 from this graph (Figs 1D and S1D). These two proteins were considered contaminants based on their function and subcellular localisation. Pathway enrichment analyses were performed using DAVID version 6.8 [32,33]. Indicated proteins were searched against a background of all human proteins quantified using default settings. A representative term was selected from each annotation cluster with a Benjamini-corrected p-value <0.05. Figures show the selected term with corresponding fold enrichment and log_10_ transformation of the Benjamini-corrected p-value. InterPro domain annotations [68] were added to proteins modulated according to ‘sensitive’ criteria (S3 Table). Putative immune ligands were defined by domain annotations cadherin, collagen, HLA, C-type lectin, immunoglobulin, Ig, TNF or butyrophylin.

### Statistical analysis

Figs 1D and 7A and S1D: p-values were estimated using significance A with Benjamini-Hochberg correction for multiple hypothesis testing [117]. For proteins quantified in both replicates, a one-way ANOVA was used to estimate p-values for FC during the infection time-course. Per replicate, the average value of mock 1.5 & 18 hpi was used as a control. P-values were corrected for multiple hypothesis testing using the Benjamini-Hochberg method. A corrected p-value of <0.05 was considered statistically significant. S1E Fig: the correlation between PMP biological replicates was determined by linear regression after plotting the log_2_ fold-change (VACV 18hpi / mock).

## Supporting information

S1 Fig Technical details of the proteomic plasma membrane profiling experiments. Related to Fig 1. (**A-B**) HFFF-TERTs were mock-treated or infected with VACV at MOI 5 in parallel with infections for PMP. At 15.5 hpi samples were fixed and stained for the late VACV protein D8 to assess infection levels. (**A**) Representative gating strategy of VACV-infected cells stained with anti-D8 followed by anti-mouse-PE or with the secondary antibody only (fluorescence minus one, FMO) as a control. Viable cells and single cells were gated followed by selection of D8-positive cells. (**B**) D8 levels in mock-treated or VACV-infected cells for each of the two biological repeats. (**C**) Correlation of protein abundance (signal: noise, S: N) of mock-treated samples at 1.5 h and 18 h per replicate. A single human protein was excluded from PMP2 because the abundance in mock samples was ‘0’. (**D**) Fold-change of VACV and human PM proteins quantified in both repeats. Scale of the x-axis was not limited (as in Fig 1D) to include VACV proteins C6 and RPO7, which are considered outliers based on their function and subcellular localisation. (**E**) Correlation of the fold-change of VACV and human PM proteins quantified at 18 hpi in PMP1, PMP2 or a third biological repeat PMP3 performed at 18h post infection for further validation of results.
(TIF)

S2 Fig Cell surface expression of NK/T cell ligands during VACV infection. Related to Fig 4. Temporal profiles of known NK/T cell ligands. (**A**) Adhesion molecules. (**B**) Plexins. (**C**) Natural cytotoxicity triggering receptor (NCR) ligands. (**D**) Apoptosis regulators. (**E**) Co-inhibitory/stimulatory molecules. (**F**) Other. Data are represented as mean ± SD (PMP n = 2, $ PMP n = 1; WCL [9] n = 3, # WCL < n = 3). (**B**) Downregulation of plexin B1 during VACV infection was confirmed by flow cytometry in HeLa cells at 15 hpi with VACV (MOI 5). Results are representative of at least 2 independent experiments.
(TIF)

S3 Fig Downregulation of human PM proteins due to high protein turnover. (**A**) Identification of human PM proteins >2-fold downregulated at 18 hpi after VACV infection (compared to 18 h mock) and for which the protein abundance decreased >2-fold in 18 h was determined in a previous pulse (p)SILAC screen in mock-treated HFFF-TERTs [30]. (**B**) Temporal profile of selected host proteins downregulated from the cell surface during VACV infection and which have a short half-life. Data are represented as mean ± SD (PMP n = 2; pSILAC n = 1 [30]).
(TIF)

S1 Table Interactive spreadsheet of all data in the manuscript. Related to Figs 2–9 & S2. The ‘Plotter’ worksheet enables the generation of graphs for all human and viral proteins quantified. The ‘Data original’ (PMP1 & 2) and ‘replicate 3 18h’ (PMP3) worksheets contain minimally annotated protein data where the raw data has been modified by formatting and normalisation. The ‘Data’ worksheet the HLA-A, -B, -C data is based only on peptides uniquely attributed to these three HLA subtypes. The spreadsheets include data obtained in previously performed WCL proteomics [9].
(XLSX)

S2 Table Plasma membrane proteins modulated during VACV infection. Related to Fig 2. (**A-D**) Human PM proteins (A/C) downregulated or (B/D) upregulated according to (A-B) ‘sensitive’ or (C-D) ‘stringent’ criteria. (**E-F**) DAVID functional enrichment analysis of proteins shown in (A) or (B), respectively, compared to all quantified human PM proteins.
(XLSX)

S3 Table Discovery of putative immune ligands using InterPro domain annotation. Related to Fig 5. InterPro functional domain annotation of proteins (A) downregulated or (B) upregulated according to ‘sensitive’ criteria.
(XLSX)

S4 Table Systematic analysis of mechanism underlying protein modulation during VACV infection. Related to Figs 7 and S3. (**A**) Human PM proteins quantified in both repeats and on average >2-fold downregulated compared to 18 h mock, in combination with p-value <0.05 and RR >1.5. (**B**) Human PM proteins quantified in both repeats and on average >2-fold upregulated compared to 18 h mock, in combination with p-value <0.05 and RR <0.66. (**C**) Human PM proteins quantified in both repeats and on average >2-fold downregulated compared to 18 h mock, in combination with p-value <0.05 and >2-fold pSILAC turnover rate in 18 h [30]. P-values on the fold-change at 18 hpi was estimated using the method of significance A and corrected for multiple hypothesis testing [117].
(XLSX)

S5 Table Comparison of protein expression at whole cell level and at the plasma membrane during VACV infection. Related to Fig 8. (**A**) Human PM proteins downregulated in both PMP and WCL experiments [9] using ‘sensitive’ criteria. (**B**) Human PM proteins downregulated in PMP but not WCL using ‘sensitive’ criteria. (**C**) DAVID functional enrichment analysis of proteins shown in (A), compared to all human PM proteins quantified in at least one replicate of PMP and WCL. (**D**) Human PM proteins upregulated in both PMP and WCL using ‘sensitive’ criteria. (**E**) Human PM proteins upregulated in PMP but not WCL using ‘sensitive’ criteria.
(XLSX)

S6 Table Human PM protein regulation by VACV and HCMV. Related to Fig 9. (**A/C**) All human PM proteins (A) downregulated or (C) upregulated >2-fold by both VACV and HCMV [29] according to ‘sensitive’ criteria. (**B/D**) DAVID functional enrichment of proteins shown in (A) and (C), respectively, compared to all proteins quantified in both VACV and HCMV PMP screens.
(XLSX)
