# Supplementary figures and images for: Selective modulation of cell surface proteins during vaccinia infection: A resource for identifying viral immune evasion strategies
Source: PLoS Pathog. 2022 Jun 21;18(6):e1010612. doi: 10.1371/journal.ppat.1010612 (PMC9307158; doi:10.1371/journal.ppat.1010612)

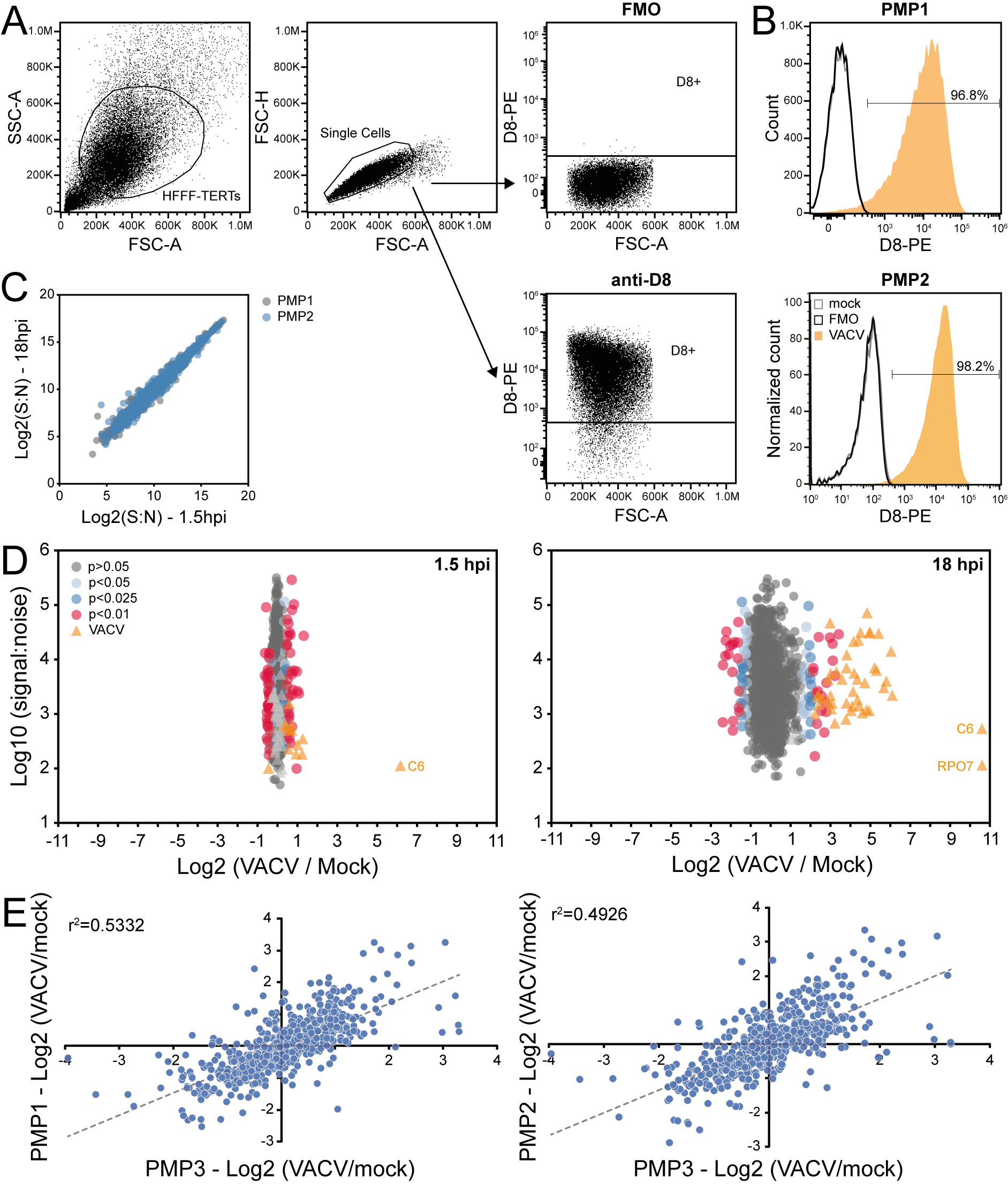

Supplement: S1 Fig — Related to Fig 1. (A-B) HFFF-TERTs were mock-treated or infected with VACV at MOI 5 in parallel with infections for PMP. At 15.5 hpi samples were fixed and stained for the late VACV protein D8 to assess infection levels. (A) Representative gating strategy of VACV-infected cells stained with anti-D8 followed by anti-mouse-PE or with the secondary antibody only (fluorescence minus one, FMO) as a control. Viable cells and single cells were gated followed by selection of D8-positive cells. (B) D8 levels in mock-treated or VACV-infected cells for each of the two biological repeats. (C) Correlation of protein abundance (signal: noise, S: N) of mock-treated samples at 1.5 h and 18 h per replicate. A single human protein was excluded from PMP2 because the abundance in mock samples was ‘0’. (D) Fold-change of VACV and human PM proteins quantified in both repeats. Scale of the x-axis was not limited (as in Fig 1D) to include VACV proteins C6 and RPO7, which are considered outliers based on their function and subcellular localisation. (E) Correlation of the fold-change of VACV and human PM proteins quantified at 18 hpi in PMP1, PMP2 or a third biological repeat PMP3 performed at 18h post infection for further validation of results. (TIF) [file ppat.1010612.s001.tif]

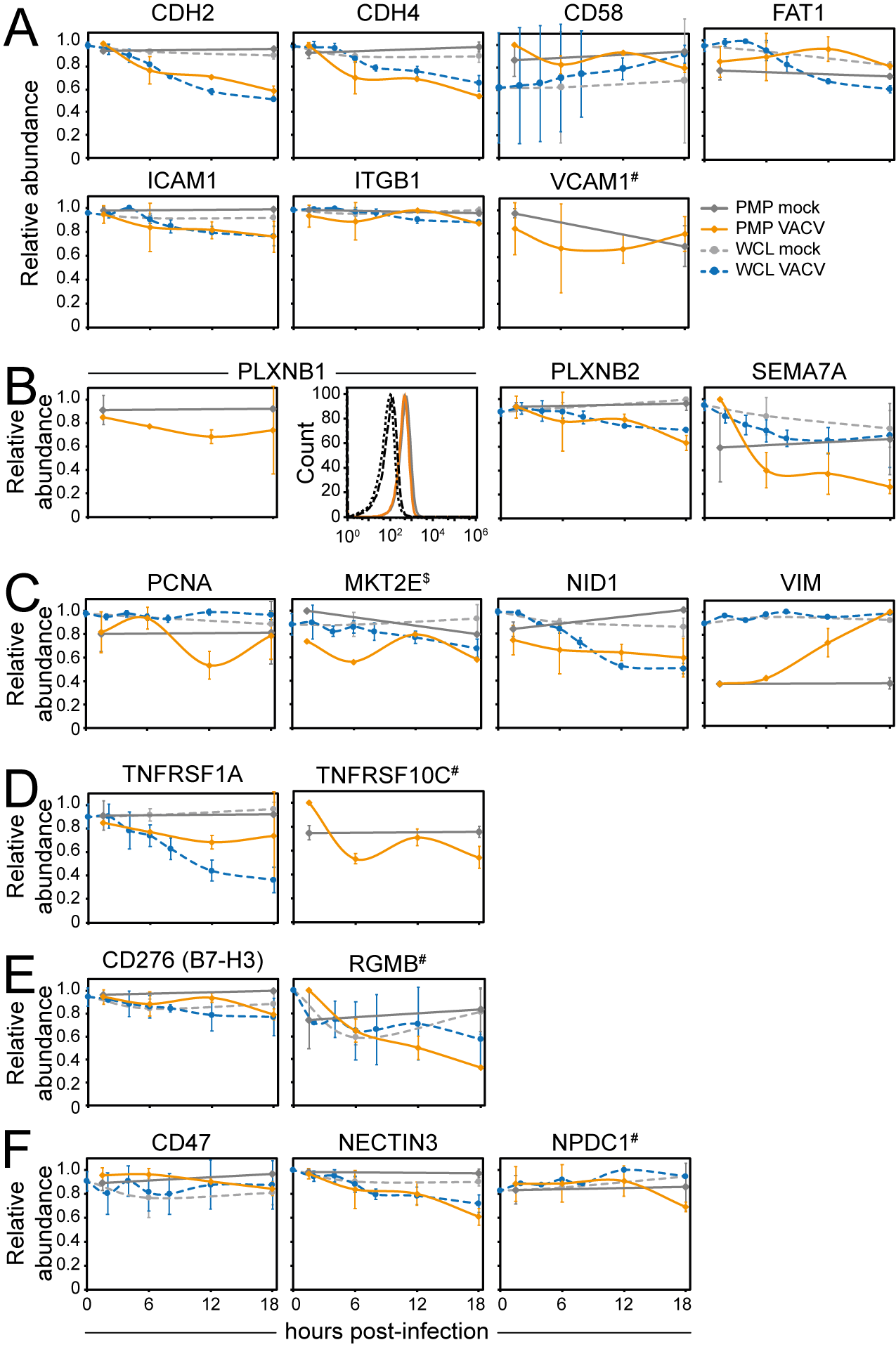

Supplement: S2 Fig — Related to Fig 4. Temporal profiles of known NK/T cell ligands. (A) Adhesion molecules. (B) Plexins. (C) Natural cytotoxicity triggering receptor (NCR) ligands. (D) Apoptosis regulators. (E) Co-inhibitory/stimulatory molecules. (F) Other. Data are represented as mean ± SD (PMP n = 2, $ PMP n = 1; WCL [9] n = 3, # WCL < n = 3). (B) Downregulation of plexin B1 during VACV infection was confirmed by flow cytometry in HeLa cells at 15 hpi with VACV (MOI 5). Results are representative of at least 2 independent experiments. (TIF) [file ppat.1010612.s002.tif]

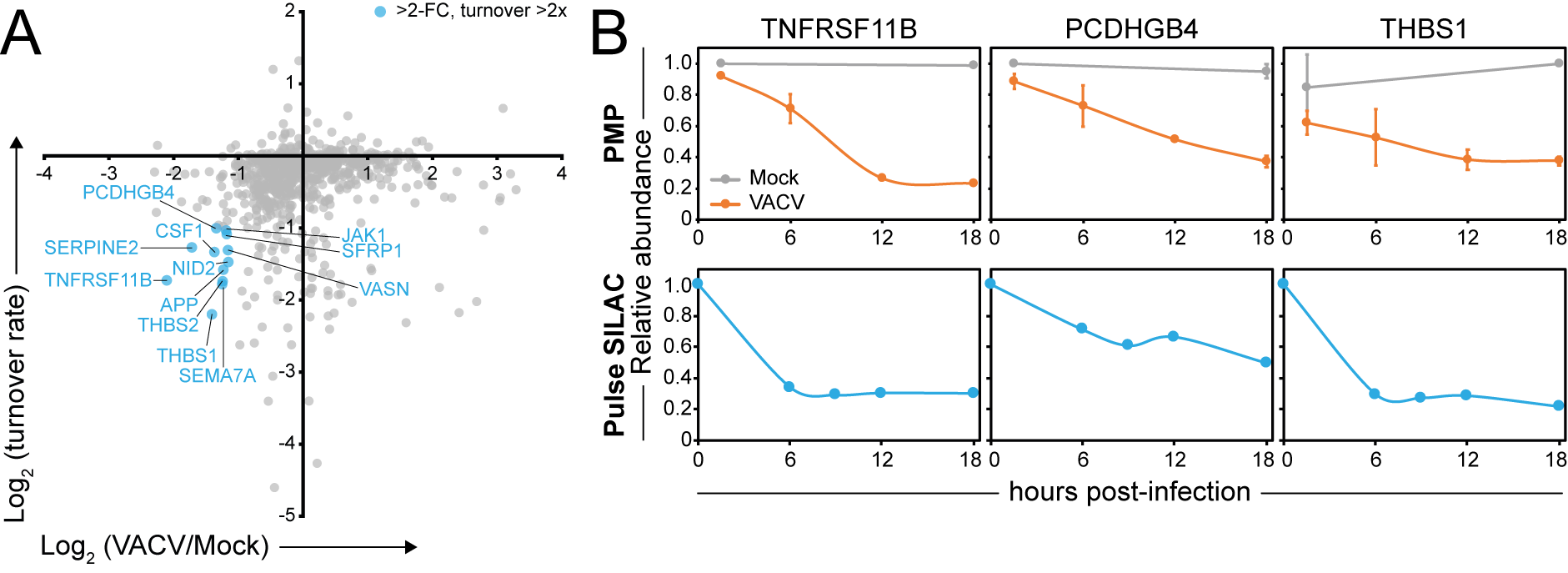

Supplement: S3 Fig — (A) Identification of human PM proteins >2-fold downregulated at 18 hpi after VACV infection (compared to 18 h mock) and for which the protein abundance decreased >2-fold in 18 h was determined in a previous pulse (p)SILAC screen in mock-treated HFFF-TERTs [30]. (B) Temporal profile of selected host proteins downregulated from the cell surface during VACV infection and which have a short half-life. Data are represented as mean ± SD (PMP n = 2; pSILAC n = 1 [30]). (TIF) [file ppat.1010612.s003.tif]
